# Anti-Mouse CD83 Monoclonal Antibody Targeting Mature Dendritic Cells Provides Protection Against Collagen Induced Arthritis

**DOI:** 10.3389/fimmu.2022.784528

**Published:** 2022-02-10

**Authors:** Pablo A. Silveira, Fiona Kupresanin, Adelina Romano, Wei-Hsun Hsu, Tsun-Ho Lo, Xinsheng Ju, Hsiao-Ting Chen, Helen Roberts, Daniel G. Baker, Georgina J. Clark

**Affiliations:** ^1^ Dendritic Cell Research, ANZAC Research Institute, Sydney, NSW, Australia; ^2^ Sydney Medical School, University of Sydney, Sydney, NSW, Australia; ^3^ Kira Biotech Pty Ltd., Brisbane, QLD, Australia

**Keywords:** CD83, dendritic cells, regulatory T cells, antigen presentation, collagen induced arthritis (CIA), mouse, monoclonal antibody

## Abstract

Antibodies targeting the activation marker CD83 can achieve immune suppression by targeting antigen-presenting mature dendritic cells (DC). This study investigated the immunosuppressive mechanisms of anti-CD83 antibody treatment in mice and tested its efficacy in a model of autoimmune rheumatoid arthritis. A rat anti-mouse CD83 IgG2a monoclonal antibody, DCR-5, was developed and functionally tested in mixed leukocyte reactions, demonstrating depletion of CD83^+^ conventional (c)DC, induction of regulatory DC (DCreg), and suppression of allogeneic T cell proliferation. DCR-5 injection into mice caused partial splenic cDC depletion for 2–4 days (mostly CD8^+^ and CD83^+^ cDC affected) with a concomitant increase in DCreg and regulatory T cells (Treg). Mice with collagen induced arthritis (CIA) treated with 2 or 6 mg/kg DCR-5 at baseline and every three days thereafter until euthanasia at day 36 exhibited significantly reduced arthritic paw scores and joint pathology compared to isotype control or untreated mice. While both doses reduced anti-collagen antibodies, only 6 mg/kg achieved significance. Treatment with 10 mg/kg DCR-5 was ineffective. Immunohistological staining of spleens at the end of CIA model with CD11c, CD83, and FoxP3 showed greater DC depletion and Treg induction in 6 mg/kg compared to 10 mg/kg DCR-5 treated mice. In conclusion, DCR-5 conferred protection from arthritis by targeting CD83, resulting in selective depletion of mature cDC and subsequent increases in DCreg and Treg. This highlights the potential for anti-CD83 antibodies as a targeted therapy for autoimmune diseases.

## Introduction

Dendritic cells (DC) are a rare heterogeneous immune population that can be broadly separated into conventional (c) and plasmacytoid (p)DC subsets. These cells constantly survey their tissue environments for antigens. Antigens are internalized, processed, and transported by DC to lymphoid organs where they are presented as peptides *via* major histocompatibility complex (MHC) molecules to T cells. On encountering antigen accompanied with inflammatory cytokines or danger signals triggering pattern recognition receptors, DC mature (1). This results in cytokine and chemokine secretion and upregulation of surface coreceptors required to activate specific T cell responses. In the absence of danger signals or inflammatory cytokines, antigen presentation by immature DC directs T cell tolerance through mechanisms such as deletion, anergy or induction of regulatory T cells (Treg) (2). Alternatively, signaling through anti-inflammatory cytokines such as IL-10, homeostatic signals through vitamin D or reverse signaling through costimulatory receptors CD80/86 guide DC to mature into a regulatory state (known as DCreg) that actively induce T cell tolerance (3–5). Inappropriate maturation of DC presenting self-antigens or ineffective induction of DCreg can lead to activation of autoreactive T cells causing autoimmune diseases (6, 7).

Rheumatoid arthritis (RA) is a debilitating systemic autoimmune disease causing inflammation, pain, and ultimately loss of joint function that affects approximately 1% of adults worldwide (8). While targeted therapies with limited immune suppression have become common and effective, therapies that target novel pathways involved in the initiation of the immune response (e.g., antigen presentation) are desirable to arrest disease at an earlier stage. Collagen induced arthritis (CIA) is a mouse model of this disease driven by mature DC in lymphoid organs and synovial fluid initiating self-reactive T cell and B cell responses through antigen presentation and coincidental inflammatory cytokine and chemokine secretion (9, 10). In arthritis mouse models, adoptive transfer of DC prevented from maturing by genetic or chemical means suppressed arthritogenic T and B cell activation by inducing Treg (11–13), a mechanism which has been translated to human clinical trials (14, 15). Targeting mature DC *in situ* with antibodies is a potential strategy for treating RA.

CD83 is an Ig-superfamily molecule most highly expressed on the surface of mature DC, but also present transiently at lower levels on other activated antigen presenting cells (APC) such as B cells and subsets of activated T cells (16, 17). CD83 contributes to APC maturation by binding and sequestering the ubiquitinase ligase MARCH1, inhibiting the ability of the enzyme to ubiquitinate and degrade surface MHC class II and CD86 molecules (18). CD83 is also released in a soluble form with demonstrated immunoregulatory properties (17). In order to therapeutically target mature DC in inflammatory diseases, our group developed antibodies to the human CD83 molecule, first as a polyclonal reagent to demonstrate proof of principle (19, 20), and then as a therapeutic affinity matured human IgG1, Fc-competent monoclonal antibody (mAb), 3C12C (21). Both reagents facilitated antibody-dependent cell cytotoxicity (ADCC) killing of CD83^+^ targets including human DC populations *in vitro*, preventing alloreactive T cell presentation and proliferation in mixed leukocyte reaction (MLR) assays. The antibodies also target DC populations in human peripheral blood mononuclear cell (PBMC) xenografted SCID mice, effectively suppressing xenogeneic T cell responses, thereby preventing graft-versus-host disease. Importantly, anti-CD83 antibodies preserved xenogeneic anti-viral and anti-tumor T cell responses and did not target xenogeneic Treg or immature DC that enhance self-tolerance (20, 21). 3C12C has shown similar functional characteristics and a good safety profile in a non-human primate model (22). Another group showed that a mouse anti-human CD83 mAb could deplete DC and restrain self-reactive T cell generation in a xenograft temporal-artery plus PBMC SCID mouse model of autoimmune vasculitis indicative of potential utility for anti-CD83 treatment in autoimmune diseases (23).

To gain a better understanding of the immunoregulatory mechanisms of anti-CD83 antibodies, we generated a rat anti-mouse CD83 mAb, DCR-5, that mimics the properties of our human anti-human CD83 therapeutic 3C12C. Using *in vitro* and *in vivo* assays, we showed that the DCR-5 antibody has the potential to deplete mature CD83^+^ cDC and induce DCreg, leading to reduced T cell activation and greater Treg induction. This immune suppressive function of DCR-5 was effective in decreasing the severity of arthritis in the CIA mouse model of disease.

## Methods

### Mice

For the CIA model, DBA/1 male mice were ordered from Envigo (Indianapolis, IN) and housed at the Bolder BioPath (Boulder, CO) facility under specific pathogen free conditions. All other mice were housed at the ANZAC Research Institute (Sydney, Australia) under specific pathogen free conditions. C57BL/6JAusb (B6), BALB/cJAusb (BALB/c), and B6.SJL-*Ptprc^a^Pepc^b^*/BoyJAusb (B6.*Ptprc^a^*) mice (24) were purchased from the Animal BioResources (Moss Vale, Australia) and DBA/1J mice were purchased from the Walter and Eliza Hall Institute (Parkville, Australia). B6.129S4-*Cd83^tm1Tft^*/J (CD83KO) mice (25) were purchased from The Jackson Laboratory (Bar Harbor, ME).

### Production of Anti-CD83 mAb

Rat hybridomas were generated by the Monash Antibody Technologies Facility (Clayton, Australia). Four Wistar rats were immunized intra-peritoneally (i.p.) three times at 2-weekly intervals with 16 µg recombinant His-tagged extracellular domain (Met 1-Arg 133) of mouse CD83 (Sino Biologicals, Beijing, China) and Sigma Adjuvant System plus methylated CpG (Sigma-Aldrich, St. Louis, MO). Splenocytes from rats were fused to the mouse myeloma cell line SP2/0-Ag14 using a conventional polyethylene glycol fusion protocol. IgG-producing hybridomas from the first two rats were selected for binding to recombinant mouse CD83-Fc (Sino Biological) by ELISA. Two additional rounds of selection were performed on lipopolysaccharide (LPS; *In vivo*gen, San Diego CA) stimulated (1 µg/ml, 4 h) A20 cell line (ATCC; Manassas, VA) by flow cytometry culminating in the DCR-3 clone. IgG hybridomas from the other two rats were selected by flow cytometry comparing binding to CRISPR/Cas9 CD83 deleted and parent WT mutuDC 1940 strain [kindly provided by Justine Mintern and Hans Acha-Orbea (26)] stimulated for 18 h with 1 µM CpG-ODN 2395 (*In vivo*gen), culminating in the selection of the DCR-5 clone. DCR-3 and DCR-5 hybridomas were adapted to serum free media (Hybridoma-SFM; ThermoFisher, Waltham, MA) before being grown in CELLine Bioreactor flasks (Wheaton, Staffordshire, UK) to produce bulk quantities of antibodies that were purified by Protein G affinity chromatography (GE Healthcare, Chicago IL) on an NGC Affinity Chromatography System (Bio-Rad, Hercules CA). Antibodies were passed through 0.22 µm filters (Interpath, Pendleton, OR) and isotypes determined by rat mAb isotyping test kit (AbD Serotec, Kidlington, UK). The average purity of antibody bands in DCR-5 preparations were determined on average to be 95.1% over multiple reads using a Bioanalyzer Agilent Protein 230 Chip (Agilent Technologies).

### Flow Cytometry

CD83 binding of purified DCR-3, DCR-5 or Michel-19 (BD Biosciences, San Jose, CA) antibodies were examined at 20 µg/ml and detected with a goat anti-rat IgG-AlexaFluor (AF)-488 secondary antibody (ThermoFisher). Equal amounts of purified rat IgG1/κ (R3-34), rat IgG2a/κ (R35-95) or rat IgG2b/κ (A95-1) isotype controls (BD Biosciences) were used for comparison. Rat serum (10%) and Fc Block (2.4G2; BD Biosciences) were used to respectively block anti-rat antibodies and Fc receptors before staining with combinations of the fluorochrome-labeled anti-mouse antibodies: CD4-Brilliant Violet (BV)650 (RM4-5), CD8a-peridinin-chlorophyll-protein (PerCP)-Cy5.5 (53-6.7), CD11b-V500 (M1/70), CD11c-phycoerythrin (PE)-Cy7 or allophycocyanin-Cy7 (N418), CD25-PE (PC61.5), CD40-PerCP-Cy5.5 (3/23), CD45.1-BV650 (A20), CD45.2-PerCP-Cy5.5 (104), CD45R/B220 (RA3-6B2)-allophycocyanin, CD80-PE-CF594 (16-10A), CD86-PE (GL-1), F4/80-fluorescein isothiocyanate (FITC) (BM8); H-2K^b^-FITC (AF6-88.5), IA/IE-PE or AF700 (M5/114-15.2), Ly-6C-PerCP-Cy5.5 (HK1.4), PDCA-1- allophycocyanin (JF05-1C2.4.1), PD-L1-BV711 (Ty75), PD-L2-allophycocyanin (10F.9G2) from BD Biosciences, ThermoFisher, BioLegend (San Diego, CA) or Miltenyi Biotec (Bergisch Gladbach, Germany). The Michel-19 anti-CD83 clone on FITC (Biolegend) or BV711 (BD Biosciences) was used to assess CD83 expression in DCR-5/isotype control treated samples. Lineage (Lin) specific biotinylated antibodies CD3e (145-2C11), CD19 (1D3), Ly6G (1A8) and NK1.1 (PK136) were detected using streptavidin-BV421 (BioLegend or BD Biosciences). Cell viability was assessed with 3 µM DAPI or Zombie Aqua (ThermoFisher) staining. After surface staining, intracellular FoxP3-allophycocyanin (FJK-16s), IDO1-AF647 (2E2/IDO1) or IL-10-BV650 (JES5-16E3) staining was performed using the Foxp3/Transcription Factor Staining Buffer Set Kit (ThermoFisher). Cells were stimulated with 50 ng/ml phorbol myristate acetate and 1 µg/ml ionomycin (Sigma Aldrich) with BD Golgi-Plug (BD Biosciences) for 4 h prior to staining. T cells were labeled with 0.5 µM Cell Trace Violet (CTV) or carboxyfluorescein succinimidyl ester (CFSE; ThermoFisher) to assess proliferation. Data was collected on a BD LSR Fortessa flow cytometer and analyzed on FlowJo 10 software (BD Biosciences).

### *In Vitro* Assays

All assays were performed in RPMI 1640 media supplemented with 10% fetal calf serum, 2 mM GlutaMAX, 100 U/ml penicillin, 100 mg/ml streptomycin, 1 mM sodium pyruvate, 10 mM HEPES and 50 mM 2-mercaptoethanol (all ThermoFisher). DC and B cell activation was achieved by culturing red blood cell depleted splenocytes (2 × 10^6^ cells/ml) in 1 µg/ml LPS or 5 µM CpG-ODN 2395 for 18 h. T cells were activated by culturing 2 × 10^5^ T cells with 1 × 10^5^ anti-CD3/28 Dynabeads (ThermoFisher) for 18 h. For DC depletion, the indicated concentrations of antibodies were added to an MLR in which 5 × 10^5^ B6 and 5 × 10^5^ BALB/c or DBA/1 splenocytes (i.e., 1:1 ratio) in the presence of 100 U/ml human IL-2 were cocultured in 96-well plates for 18 h. The T cell proliferation MLR assay was performed as above with CTV labeled B6.*Ptprc^a^* splenocytes, with cells cultured for 84 h. Purified low-endotoxin azide-free rat IgG2a/κ (R35-95) or rat IgG2b/κ (A95-1) mAb (BD Biosciences) were used as isotype controls. The mouse T helper cell cytometric bead array (BD Biosciences) was used to measure cytokines in MLR supernatants using manufacturer’s instructions. For Treg suppression assay, CD4^+^ CD25^+^ T cells were purified by MACS from spleens of B6 mice treated i.p. with 150 µg DCR-5 or anti-trinitrophenol rat IgG2a/κ mAb (InVivoPlus 2A3, BioXcell, Lebanon NH) isotype control following the CD4^+^CD25^+^ regulatory T cell isolation kit instructions (Miltenyi Biotec). FoxP3 was confirmed on 94–96% of isolated cells from both strains. The CD4^+^CD25^-^ conventional T cell (Tcon) fraction was purified from spleens of untreated B6.*Ptprc^a^* mice using the same kit and labelled with 0.5 µM CFSE. Labeled Tcon (2.5 × 10^4^) were cultured with DCR-5 and isotype control treated Tregs at the indicated ratios with 12.5 × 10^4^ mouse T activator CD3/CD28 Dynabeads (ThermoFisher). CD45.1^+^CD4^+^ Tcon proliferation was assessed by CFSE dilution after 3 days using flow cytometry. Flt-3L cultured bone marrow DC (FL-DC) were generated by culturing B6.*Ptprc^a^* bone marrow with complete RPMI containing 12.5% B16 cell line supernatant as described (27). DC (2.5 × 10^6^ cell/ml) were washed and re-cultured with 20 µg/ml of DCR-5 or rat IgG2a/κ mAb (InVivoPlus 2A3, BioXcell) isotype control in complete RPMI overnight to assess DC surface marker expression. To assess Treg induction, antibody treated DC were washed and then re-cultured for 84 h with 1 × 10^5^ BALB/c T cells (0.5 µM CFSE labeled for proliferation or non-labeled for intracellular staining) at a 1:8 ratio.

### *In Vivo* Assays

B6 or DBA/1 mice were injected i.p. with 200 µl PBS containing the indicated amount of DCR-5, rat IgG2a (InVivoPlus 2A3, BioXcell) isotype control or untreated. Mice were euthanized for analysis when indicated.

### Immunoprecipitation and Immunoblot

B6 FL-DC stimulated with 1 µg/ml LPS overnight were washed and incubated with a 1 mg/ml sulfo-N-hydroxysulfosuccinimide (NHS) biotin in PBS (pH 8.0) solution. The biotinylation reaction was inactivated by washing the cells with 100 mM glycine in PBS before being lysed in mammalian protein extraction reagent (ThermoFisher). Protein A conjugated Dynabeads (ThermoFisher) coated with 10 µg rat IgG2a isotype control (2A3, BioXcell) were incubated with the lysate to clear it of non-specific binding proteins. The beads were removed using a magnetic column. Dynabeads coated with 10 µg purified DCR-5 or Michel-19 (BD Biosciences) antibody were then used to immunoprecipitate CD83 from lysate. Target protein was eluted reduced and denatured prior to being run on a 4–12% Bis-Tris Plus gel (ThermoFisher) and transferred to nitrocellulose membrane using the iBlot system (ThermoFisher). Membranes were blocked with 5% skim milk powder in TBST and incubated with Streptavidin-HRP (BioLegend) to detect biotinylated cell surface derived proteins. Alternatively, recombinant His-Tag mouse CD83 or CD302 (Sino Biological) were immunoprecipitated with DCR-5 or Michel-19 coated Dynabeads and immunoblotted with anti-His Tag-HRP (BioLegend). Signal was detected with an ECL reagent (Clarity ECL kit; Bio-Rad) and visualized using the Chemidoc image system (Bio-Rad). SeeBlue protein standards (ThermoFisher) were used for size comparison.

### CIA Model

The CIA model was performed by Bolder BioPath. DBA/1 male mice immunised intradermally into the tail base with 200 µg bovine type II collagen (Bolder BioPath) and 250 µg complete Freunds’ adjuvant (MP Biomedical, Irvine CA). I.p. treatment with the indicated concentrations of DCR-5 or isotype control was initiated on d0 and continued every 3 days. Control groups included naïve non-immunized mice or immunized mice that were either untreated or treated with dexamethasone (0.2 mg/kg; VetOne, Boise ID) subcutaneously every second day. Body weights (d0-36) and mean clinical scoring of four paws (d21-36) were recorded: 0 = Normal; 1 = 1 hind or fore paw joint affected or minimal diffuse erythema and swelling; 2 = 2 hind or fore paw joints affected or mild diffuse erythema and swelling; 3 = 3 hind or fore paw joints affected or moderate diffuse erythema and swelling; 4 = marked diffuse erythema and swelling, or 4 digit joints affected; 5 = severe diffuse erythema and severe swelling of the entire paw, unable to flex digits. On d36, animals were anesthetized and exsanguinated followed by cervical dislocation to collect spleen, paw and knee tissues for histological analysis. Timed euthanasia was performed on an additional untreated group, with two mice euthanized pre-immunization and remaining mice euthanized at days 1, 2, 9, 22, and 36 post-immunization.

### Histology

Histopathologic evaluation of knee and paw tissue from CIA studies were performed by HistoTox Labs (Boulder, CO). Tissues were fixed 1–2 days in 10% formalin and decalcified 4–5 days in 5% formic acid before being embedded in paraffin. Sections were stained with toluidine blue and images were acquired using a Axioscan Z1 slide scanner and analysed using Zen software (Zeiss, Oberkochen, Germany). Inflammation, pannus formation, cartilage damage, bone resorption, and periosteal new bone formation were scored (0–5) in a blinded fashion as described (28). OCT snap frozen spleen sections were dried overnight, fixed with ice-cold acetone, and blocked with 1% BSA in PBS before staining. For immunofluorescence, spleen sections were incubated with biotinylated anti-CD83 (Michel-19; BioLegend) and subsequently with streptavidin-AF488 (ThermoFisher), anti-CD11c-AF594 (N418; BioLegend) and anti-CD45R-AF647 (RA3-6B2; BioLegend), washing with 0.5% BSA in PBS in between. For immunohistochemistry, endogenous peroxidase in spleen sections was quenched with 0.3% H_2_O_2_/PBS. The sections were blocked with an Avidin/Biotin blocking kit as per manufacturer’s instructions (Vector Labs, Burlingame CA) and then individually stained with biotinylated CD11c (N418; BioLegend), FoxP3 (FJK-16s; ThermoFisher) or PBS alone, detected using a Streptavidin-HRP (BioLegend) secondary and DAB Peroxidase Substrate (Vector Lab, Burlingame, CA). Each section was finally counterstained with 10% Harris hematoxylin solution (Australian Biostain, Traralgon, Australia). Tissue sections were imaged on an EVOS-FL II Cell Imaging System (ThermoFisher) and then processed and analyzed with Image J software (NIH).

### ELISA

For anti-CD83 ELISAs, plates were coated overnight with 1 µg/ml mouse CD83-Fc (Sino Biologicals) and blocked with 5% BSA. Plates were overlayed with rat anti-mouse CD83 antibody, 1:100–1:1,000 diluted serum or hybridoma supernatant (50 µl) and then an HRP conjugated goat anti-rat IgG Fc-specific antibody (Sigma-Aldrich). The HRP substrate SIGMAFAST OPD solution (Sigma-Aldrich) was used to detect binding of antibodies by reading absorbance at 450 nm on a Victor3 Multilabel Plate Reader (Perkin Elmer). Wells were washed with 0.05% Tween 20 in PBS between steps. To detect anti-DCR-5 antibodies, ELISA plates were coated with 10 µg/ml DCR-5 and blocked with 5% BSA. Plates were overlayed with serum samples diluted 1:10,000 and 1:100,000 in PBS, without incubation (no spike) or after incubation for 30mins with 10µg/ml DCR-5 (spike). Bound mouse antibodies were detected with HRP conjugated pre-adsorbed goat anti-mouse IgG (ab97040, Abcam, Cambridge, MA), measured as above. Anti-collagen antibodies produced in the CIA model were measured in 1:500 and 1:40,000 diluted serums using the mouse anti-type I/II collagen IgG assay kit with TMB (Chondrex, Redmond WA) under manufacturers’ instructions. The mouse IL-10 ELISA (ThermoFisher) performed on FL-DC supernatants was conducted using the manufacturer’s instructions.

### Statistics

Statistical tests include t-tests or one and two-way ANOVA for comparison of two or multiple groups, respectively, as indicated in figures and performed in GraphPad Prism (San Diego, CA). Correlation was assessed by calculating Pearson’s correlation coefficient. Error bars mark standard error of the mean.

## Results

### Development of Novel Rat Anti-Mouse CD83 mAbs

**Figure 1:**
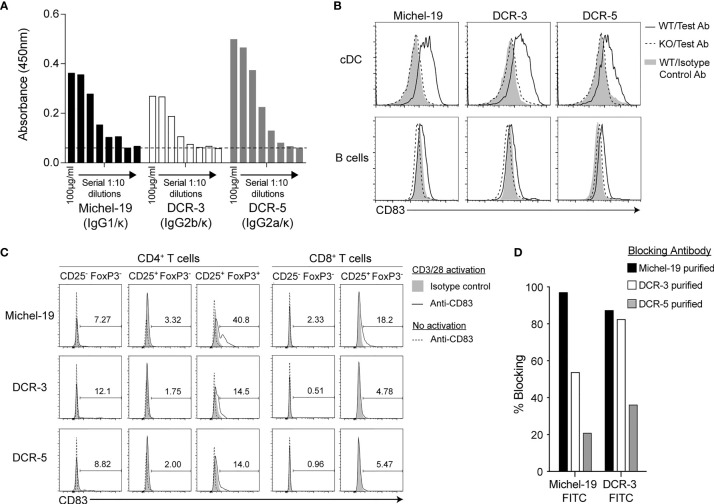
Binding properties of rat anti-mouse CD83 antibodies DCR-5 and DCR-3. **(A)** Comparison of binding of DCR-5, DCR-3 and Michel-19 anti-CD83 antibody clones (1:10 serial dilutions starting at 100 µg/ml) to plates coated with recombinant mouse CD83-His by ELISA. Dashed line marks the absorbance in the no antibody control. Binding of 20 µg/ml Michel-19, DCR-3 or DCR-5 antibody (or respective isotype controls) was compared on the surface of **(B)** gated cDC and B cells (see **Supplementary Figure 1A** for gating strategy) in WT and CD83KO splenocytes cultured overnight with 1 µg/ml LPS or; **(C)** gated FoxP3^-^CD25^-^ (naïve) and CD25^+^ (activated) CD8^+^ T cell cells and FoxP3^−^CD25^−^ (naïve), FoxP3^−^CD25^+^ (activated) and FoxP3^+^CD25^+^ (Treg) CD4^+^ T cell subsets cultured with or without overnight stimulation with CD3/CD28 microbeads. Primary antibodies were detected using an anti-rat IgG (Fc-specific)-AF488 antibody *via* flow cytometry. Percentage of CD83^+^ cells in CD3/28 stimulated cultures shown. No CD8^+^CD25^+^ T cells detected in unstimulated cultures. **(D)** Binding of sub-saturating concentrations of FITC conjugated Michel-19 or DCR-3 to LPS stimulated A20 cells by flow cytometry was compared with and without initial blockade with saturating concentrations of purified Michel-19, DCR3 or DCR-5. Degree of blocking was calculated as percentage reduction in MFI with 0% indicating no blocking (MFI equivalent to anti-CD83 FITC antibody alone) and 100% indicating full blocking (MFI equivalent to binding of isotype-FITC antibody).

Two hybridoma clones producing novel rat mAbs to mouse CD83 were selected from independent fusions and named DCR-3 (IgG2b/κ isotype) and DCR-5 (IgG2a/κ). Both bound similarly to mouse CD83-Fc coated ELISA plates compared to the commercial rat anti-mouse CD83 (IgG1/κ isotype) clone, Michel-19 (**Figure 1A**). Flow cytometry showed significant binding of Michel-19, DCR-3 and DCR-5 to native CD83 on the surface of splenic conventional (c)DC in WT but not CD83KO B6 splenocytes activated *in vitro* with LPS overnight (**Figure 1B**). Reduced binding of all three antibodies was seen on LPS-activated splenic B cells compared to splenic DC from WT mice. Comparison of binding to different APC subsets activated using LPS or CpG-ODN 2395 showed highest binding of Michel-19, DCR-5 and DCR-3 to activated CD8^+^ cDC (DC1) followed by CD8^-^ (CD11b+) cDC (DC2) (**Supplementary Figures 1A, B**). Lower levels of binding were found on LPS or CpG-ODN 2395 stimulated macrophages, monocytes, B cells, and plasmacytoid (p)DC. Minimal CD83 levels were detected on unactivated subsets. Similar binding profiles by these mAbs were seen on LPS activated cDC and B cells from DBA/1 (**Supplementary Figure 1C**) and BALB/c mice (not shown). DCR-3/5 did not bind to conventional CD4^+^ or CD8^+^ T cell populations that were unactivated or after 18 h of CD3/CD28 stimulation, while Michel-19 showed low level binding to activated CD25^+^CD8^+^ T cells (**Figure 1C**). Michel-19 also exhibited binding to the surface of ~40% of CD4^+^CD25^+^FoxP3^+^ Treg after CD3/28 stimulation, consistent with previous mouse studies (16, 29), but DCR-3 and DCR-5 displayed lower binding to this population. Cross-blocking studies on the LPS activated A20 B cell line showed that Michel-19 and DCR-3 inhibited each other’s binding to CD83 indicative of proximal epitopes, whereas DCR-5 minimally blocked DCR-3 or Michel-19 binding suggesting that it bound a distinct epitope (**Figure 1D**). DCR-5 and Michel-19 both immunoprecipitated recombinant His-tagged mouse CD83 protein, which was detected in an immunoblot as a ~35 kDa protein (**Supplementary Figure 1D**). Various CD83 protein bands (33, 42, 50, 57, 68, 77, and 105 kDa) were immunoprecipitated from the biotinylated cell surface of LPS stimulated mouse FL-DC (**Supplementary Figure 1E**). Corresponding bands were isolated with Michel-19, with less efficiency. Three of these bands (50, 57, and 77kDa) were immunoprecipitated from the cell surface of LPS stimulated A20 cells using DCR-5 and Michel-19 (data not shown). The assortment of CD83 bands detected by DCR-5 and Michel-19 mirrored the multiple CD83 isoforms detected by anti-human CD83 antibodies HB15a, HB15e, and 3C12C in immunoblots (16) and likely represent variations in glycosylation or splice variants of CD83.

### DCR-5 Depletes CD83^+^ cDC, Induces DCreg and Inhibits CD4^+^ T Cell Proliferation in MLR Assays

**Figure 2:**
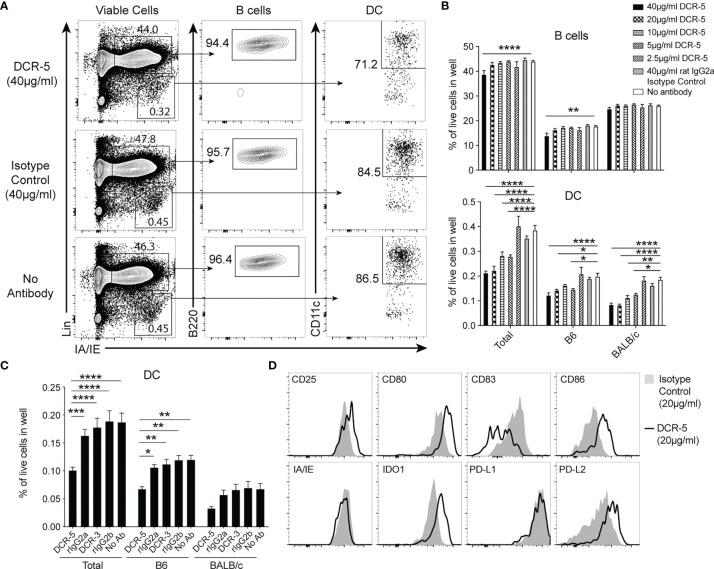
DCR-5 depletes and alters DC phenotype *in vitro*. **(A–D)** B6 and BALB/c splenocytes were cocultured overnight in MLR at a 1:1 ratio with the indicated concentrations of DCR-5 or isotype control. Levels of DC and B cells per well were determined by flow cytometry. **(A)** Representative plots showing total DC and B cells in specified groups and **(B)** mean levels of total, B6 (H-2Kd^−^) and BALB/c (H-2Kd^+^) derived cells from triplicate wells. Statistical comparisons to no antibody group by 2-way ANOVA. **(C)** Mean percentage of DC in 12 wells from 3 experiments treated with no antibody or 10 µg/ml DCR-5, DCR-3 or isotype controls. Statistical comparisons by 2-way ANOVA. *p < 0.05, **p < 0.01, ***p < 0.001 and ****p < 0.0001. **(D)** One of three representative plots of designated surface and intracellular DCreg markers expressed by total DC after overnight MLR culture with 20 µg/ml DCR-5 or isotype control.

To determine the functional activity of DCR-3 and DCR-5, their effects on activated APC and allogeneic T cell proliferation were examined when added to MLR between B6 and BALB/c splenocytes. After 18 h of culture, surface CD83 expression was observed highest on B6 and BALB/c cDC, at lower levels on B cells, but not on T cells within the MLR (**Supplementary Figure 2A**). The addition of 5–40 µg/ml DCR-5 antibody to cultures produced a significant decrease in total, B6 and BALB/c cDC, with the highest concentration causing 40–45% reduction compared to isotype control or untreated cells (**Figures 2A** **, B**). In contrast, B cells in the MLR were minimally affected by DCR-5, with only the highest dose causing a decrease of 12%. When DCR-5 and DCR-3 were compared side by side at 10 µg/ml in MLR experiments, only the former depleted cDC (**Figure 2C**), with neither affecting B cells (not shown). The DC remaining in MLR following overnight DCR-5 treatment displayed low to no surface CD83 compared to DC in isotype control treated wells. In addition, DCR-5 treatment was associated with a small reduction in surface MHC class II together with increased CD80 and CD86 costimulatory molecules in DC compared to controls, and notably augmented expression of DCreg associated surface markers CD25 and PD-L2, and the intracellular marker IDO1 (**Figure 2D**). Similar depletion of cDC (and not B cells) and induction of CD80^hi^/86^hi^ DC was observed when using DCR-5 in a B6 × DBA/1 strain MLR (**Supplementary Figure 2B**).

**Figure 3:**
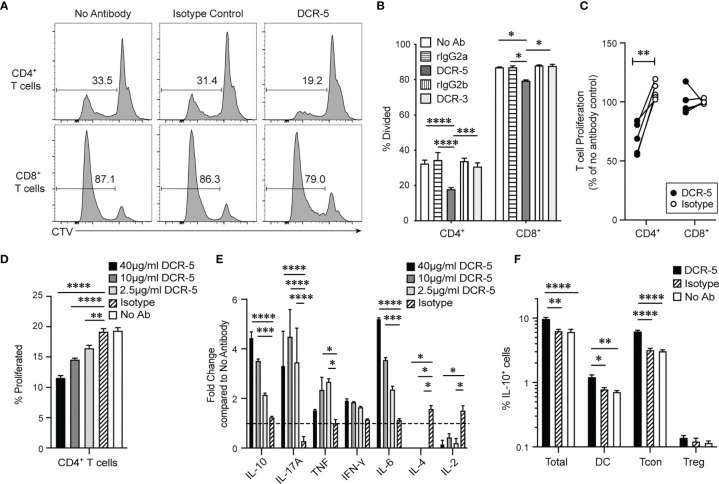
DCR-5 prevents T cell proliferation *in vitro*. BALB/c and CTV-labeled B6 splenocytes were cocultured for 84 h in MLR at a 1:1 ratio with 20 µg/ml DCR-5, DCR-3, isotype controls or no antibody. Proliferation of gated CD3^+^CD4^+^ or CD8^+^ B6 T cells was determined by CTV dilution. **(A)** Representative histograms showing % divided CD4^+^ and CD8^+^ T cells in indicated groups. **(B)** Mean levels of division in triplicate wells. Statistical comparison was determined using 2-way ANOVA. **(C)** Summary of five MLR experiments where T cell proliferation is represented as % of no antibody control. Statistical comparison was determined using a paired T-test. **(D)** CD4^+^ T cell proliferation and **(E)** cytokines in supernatant of B6 × BALB/c MLR treated with the indicated DCR-5 concentrations or 40 µg/ml isotype control after 84 h (n = 3). Cytokine data is shown as fold change compared to mean of no antibody control (dashed line). **(F)** Percentage of total cells, CD3^+^CD4^+^FoxP3^−^ (Tcon) CD3^+^CD4^+^FoxP3^+^ (Treg) T cells and CD11c^+^ IAIE^+^ (DC) that stained IL-10^+^ by intracellular flow cytometry staining after 48 h culture of B6 × BALB/c MLR with 20 µg/ml DCR-5, isotype control or no antibody. Statistical comparison to isotype control was assessed by one-way ANOVA. *p < 0.05, **p < 0.01, ***p < 0.001 and ****p < 0.0001.

DCR-3 and DCR-5 were evaluated for their effect on allogeneic T cell proliferation after 84 h in the B6 × BALB/c MLR. Adding 20 µg/ml DCR-5 reduced B6 CD4^+^ T cells undergoing proliferation compared to the isotype control (**Figures 3A** **, B**) by an average of 36 ± 5.1% over five experiments (**Figure 3C**). DCR-3 failed to reduce B6 CD4^+^ T cell proliferation and neither DCR-5 nor DCR-3 significantly affected B6 CD8^+^ T cell proliferation in the MLR (**Figures 3A** **–C**). Therefore, targeting CD83 expressing cDC in MLR cultures reduced their ability to drive CD4^+^ T cell proliferation.

We examined CD4^+^ T cell associated cytokines in the supernatants of B6 × BALB/c T cell MLR treated with 2.5, 10, and 40 µg/ml DCR-5. These doses were confirmed to significantly decrease CD4^+^ T cell proliferation in a dose dependent fashion compared to isotype and no antibody controls in the same assays (**Figure 3D**). The cytokines showing largest fold increase in DCR-5 treated supernatant were IL-10, IL-6, and IL-17A cytokines, with only IL-10 and IL-6 exhibiting a dose dependent response (**Figure 3E**). IFN-γ displayed a non-significant increasing trend with increasing doses of DCR-5 while TNF was only modestly induced by 2.5 and 10 µg/ml of DCR-5. IL-2 and IL-4 cytokines were both significantly inhibited by addition of DCR-5. The increase in immunoregulatory IL-10 and inhibition of mitogenic IL-2 are two possible factors that contribute to the decrease in CD4^+^ T cell proliferation in DCR-5 treated MLR. Intracellular staining of cells 48 h into MLR showed that the source of increased IL-10 production from DCR-5 treatment came primarily from Tcon and DC, but not Treg (**Figure 3F**).

### *In Vivo* DCR-5 Administration Reduces CD83^+^ DC and Induces DCreg and Treg in Mice

**Figure 4:**
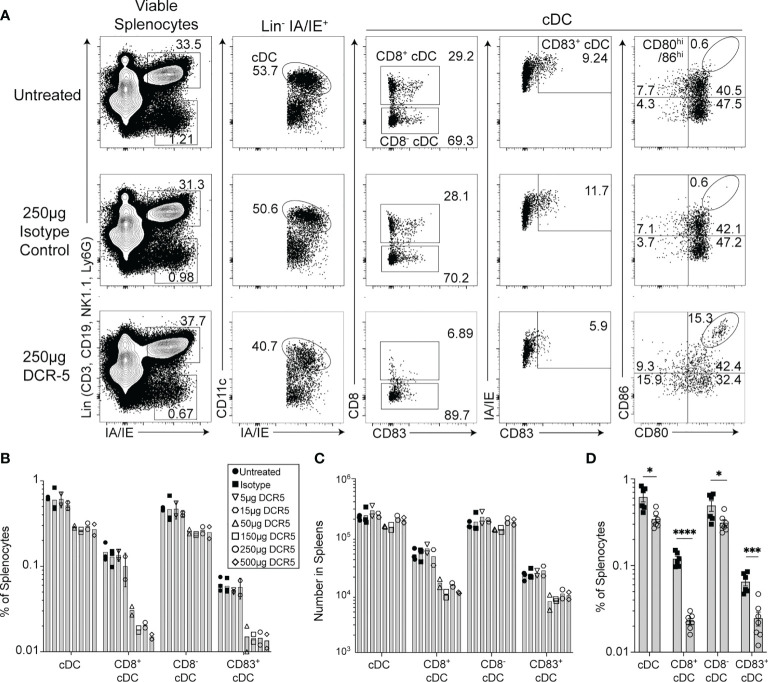
*In vivo* treatment of mice with DCR-5 results in depletion of DC. B6 mice treated with a single i.p. injection of increasing doses of DCR-5 mAb or 250 µg rat IgG2a isotype control were analyzed 48 h later for DC depletion and Treg induction by flow cytometry. **(A)** Flow cytometry plots showing splenic DC populations in untreated, 250 µg DCR-5 and 250 µg isotype control treated mice. The Lin^+^/IAIE^+^ gate shown contains >98% CD45R^+^ B cells (**Supplementary Figure 1A**). Graphs showing **(B)** frequency and **(C)** total numbers of DC populations in spleens of mice of all treatment groups. *P <*0.001 variance for treatment, 2-way ANOVA. **(D)** Combined data from four experiments showing frequency of DC populations in B6 mice treated with 250 µg DCR-5 or isotype control. Statistical comparison was determined using T-tests. *p < 0.05, ***p < 0.001 and ****p < 0.0001.

**Figure 5:**
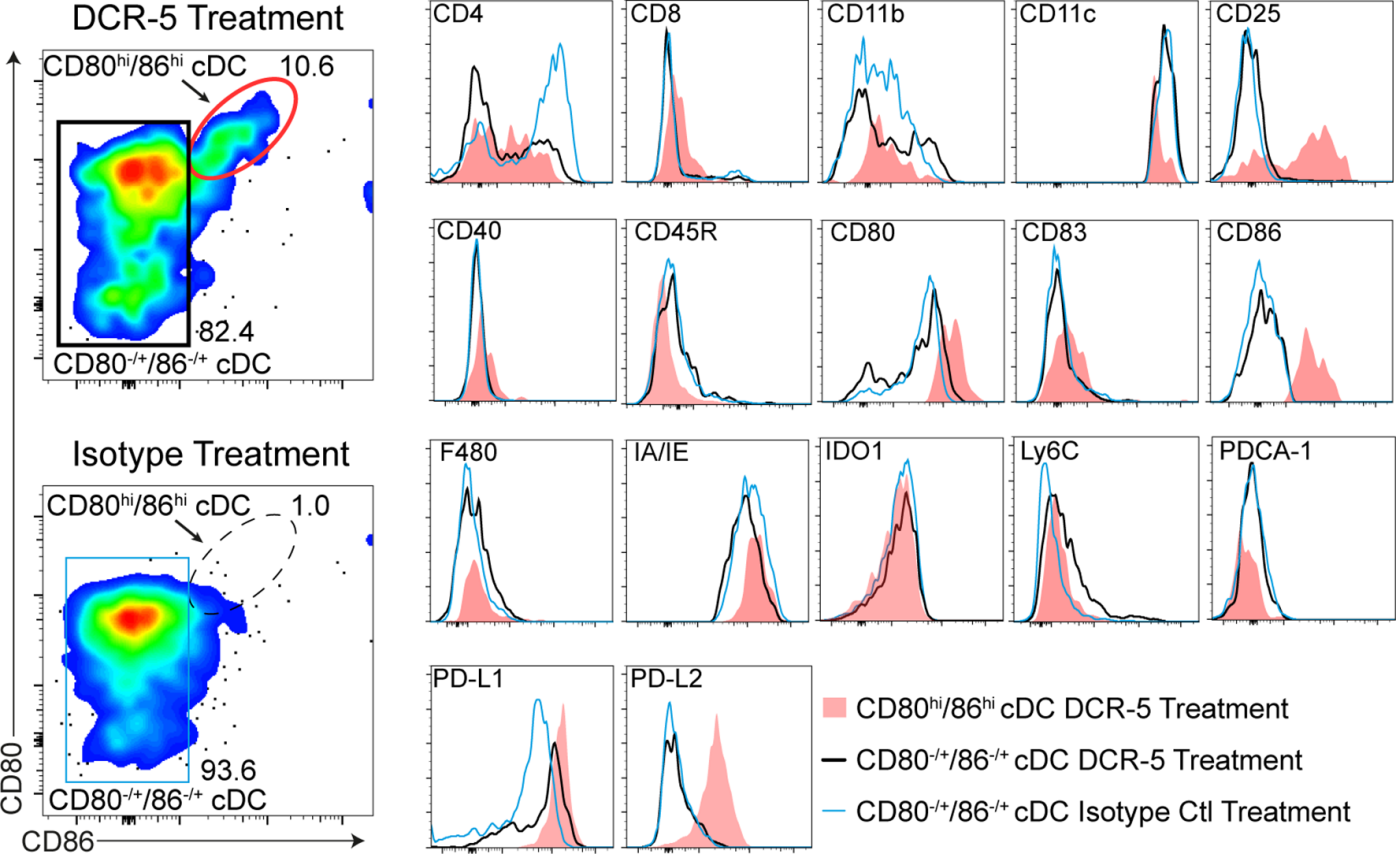
Phenotype of CD80^hi^CD86^hi^ DC population induced by DCR-5 *in vivo*. Flow cytometric expression of additional markers (histograms on right) on the populations gated in the left plots including the CD80^hi^/CD86^hi^ cDC subset induced after 48 h i.p. treatment with 250 µg DCR-5 antibody (red oval gate) compared to remaining CD80^±^/CD86^±^ splenic cDC in DCR-5 (black rectangular gate) or isotype control (blue rectangular gate) treated mice.

The *in vitro* effects of DCR-5 in the MLR assays led us to examine DC targeting *in vivo*. Eight-week-old female B6 mice were injected i.p. with varying doses of DCR-5 ranging from 5 to 500 µg. Analysis of splenocytes by flow cytometry 48 h later revealed no significant effects on pDC or B cells with any dose of DCR-5 compared to isotype control or untreated animals (**Figure 4A** and **Supplementary Figures 3A, B**). However, a small decrease in frequency, but not total numbers, of cDC was seen in mice treated with ≥ 50 µg DCR-5 (**Figures 4A** **–D**). A more prominent decrease was seen in the frequency (71–73% decrease) and number (49–63% decrease) of CD83^+^ MHCII^hi^ cDC in spleens (**Figures 4A** **–D**). It was also evident that CD8^+^ cDC had comparatively higher depletion following DCR-5 injection than CD8^−^ cDC. DCR-5 treated mice showed an increase in CD80^−/lo^/CD86^−/lo^ cDC and overall decreases in CD80^+^ cDC ± CD86 compared to isotype control and untreated mice (**Figure 4A** and **Supplementary Figure 3C**). Interestingly, like the MLR, DCR-5 treatment induced a population of CD80^hi^/86^hi^ cDC (see oval in **Figure 4A**) not present in isotype or untreated controls. This population expressed low CD11c and were CD83^dim^ compared to the remainder of cDC in DCR-5 and those in isotype treated mice (**Figure 5**). An analysis of additional markers showed that the induced CD80^hi^/86^hi^ subset did not express markers of pDC (PDCA-1, CD45R), macrophages (F4-80), monocytes (Ly6C) or innate lymphoid cells (CD117, Sca-1, CD90; not shown), but most increased CD25, PD-L1 and PD-L2 expression associated with DCreg differentiation (30). IDO1 expression was similarly expressed between CD80^hi^/86^hi^ cDC and those expressing lower CD80/86 levels in DCR-5 and isotype control treated mice. DCR-5 injected into DBA/1 mice showed a similar capacity to deplete CD83^+^ cDC and induce a CD80^hi^/86^hi^ cDC population (**Supplementary Figure 3D**).

**Figure 6:**
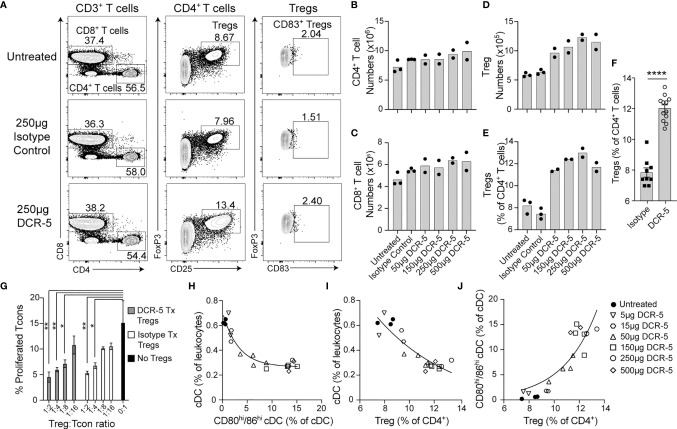
*In vivo* treatment of mice with DCR-5 results in induction of Treg. B6 mice treated with a single i.p. injection of increasing doses of DCR-5 mAb or 250 µg rat IgG2a isotype control were analysed 48 h later for T cell subsets by flow cytometry. **(A)** Flow cytometry plots in one experiment showing T cell populations in spleens of untreated, 250 µg DCR-5 and isotype control mice. Total numbers of splenic **(B)** CD4^+^ T cells, **(C)** CD8^+^ T cells and **(D)** Tregs and **(E)** Treg as a percentage of CD4^+^ T cells in mice treated with increasing DCR-5 doses. No significant difference for CD4 or CD8 T cell numbers and p <0.0001 for Tregs numbers and proportion in combined DCR-5 versus isotype treated or untreated mice (one-way ANOVA). **(F)** Combined data from five experiments showing increase in Treg (as percentage of CD4^+^ T cells) in mice treated with 150–250 µg DCR-5 or isotype control. Statistical comparison was determined using a T-test. ****p < 0.0001. **(G)** Treg suppression assay showing proliferation of CFSE labeled CD45.1^+^CD4^+^ Tcon purified from B6.*Ptprc^a^* mouse spleens cocultured with anti-CD3/28 beads and the indicated ratios of CD4^+^CD25^+^ Tregs isolated from spleens of B6 mice after 48h treatment with 150 µg DCR-5 or isotype control i.p. All groups (n = 3) compared to Tcon only group by one-way ANOVA. *p < 0.05 and **p < 0.01. Correlation of **(H)** total cDC vs. CD80^hi^/86^hi^ cDC, **(I)** total cDC vs. Treg and **(J)** CD80^hi^/86^hi^ cDC vs. Treg frequencies in spleens of DCR-5 treated mice. All correlations were *p <*0.0001 (Pearson correlation coefficient). Non-linear regression exponential curve fits shown.

We next determined whether targeting CD83^+^ cDC with DCR-5 would alter the balance of regulatory to conventional T cells in mice. No differences were seen in frequency (**Figure 6A**) or total numbers (**Figures 6B** **, C**) of splenic CD4^+^ or CD8^+^ T cells in mice treated with 50 to 500 µg of DCR-5 compared to control mice. However, DCR-5 treated mice exhibited between a 1.5- and 1.9-fold increase in splenic CD4^+^CD25^+^FoxP3^+^ Treg numbers (**Figure 6D**) and 1.5–1.7-fold increase as a proportion of CD4^+^ T cells compared to control groups (**Figures 6E** **, F**). No CD83 expression was detected on Treg of DCR-5, isotype treated or untreated mice (**Figure 6A**), suggesting that the DCR-5 was not expanding Treg through direct binding to this population. This was consistent with *in vitro* experiments demonstrating minimal binding of DCR-5 to Treg (**Figure 1C**). While present in higher numbers, Tregs isolated from mice 48 h after DCR-5 treatment exhibited comparable capacity to prevent CD25^−^ T con proliferation than those from isotype treated mice when seeded at similar Treg : Tcon ratios in a Treg suppression assay (**Figure 6G**).

Correlation analyses of B6 mice treated *in vivo* with different doses of DCR-5 demonstrated significant negative correlations (p <0.0001, Pearson coefficient correlation) between total cDC vs. CD80^hi^/86^hi^ cDC (**Figure 6H**) and total cDC vs. Treg (**Figure 6I**), while a significant positive correlation was observed between CD80^hi^/86^hi^ cDC vs. Treg (p <0.0001, **Figure 6J**). These indicate an association between these parameters.

**Figure 7:**
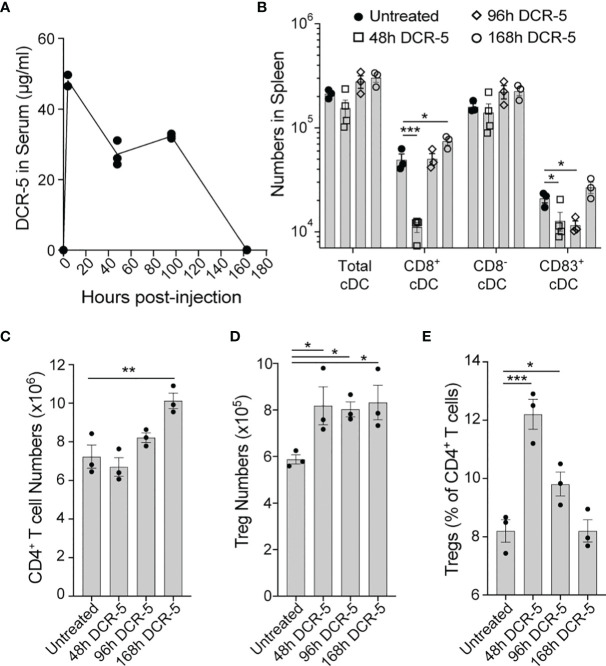
Longevity of *in vivo* DCR-5 effects on DC and Treg. B6 mice were treated with a single i.p. injection of 150 µg of DCR-5 mAb. At the indicated timepoints post-injection, analysis of **(A)** DCR-5 concentration in serum by ELISA and total numbers of splenic **(B)** DC populations, **(C)** CD4^+^ T cells, **(D)** Treg and **(E)** Treg as a percentage of CD4^+^ T cells by flow cytometry was performed. Statistical comparison to untreated group was determined using one way ANOVA. *p < 0.05, **p < 0.01 and ***p < 0.001.

The concentration of the DCR-5 antibody was measured in the serum of B6 mice at 4 h, 2 d, 4 d and 7 d after 150 µg i.p. injection (**Figure 7A**). An initial serum concentration of 48 µg/ml at 4 h post-dose, decreased to approximately half this level between 2 and 4 d, and was no longer detectable at 7 d. Reduction of splenic cDC, particularly CD8^+^ cDC, was seen after 2 d DCR-5 treatment, but returned to numbers similar to untreated mice after 4 d (**Figure 7B**). However, a decrease in CD83^+^ cDC were still seen up to 4 d. Concomitantly, the CD80^hi^/86^hi^ DC population induced by DCR-5 increased after 2 d but returned to near baseline levels by 4 d (**Supplementary Figure 3E**). Increases in splenic Treg numbers were still observed 7 d after DCR-5 treatment, however their proportion compared to conventional CD4^+^ T cells was diminished over 4–7 d post-treatment due to the increase in non-Treg (**Figures 7C** **–E**). In conclusion, *in vivo* DCR-5 treatment targets cDC populations, decreasing CD8^+^ and CD83^+^ subsets and induces maturation of a CD80^hi^/86^hi^ DCreg-like population, resulting in an increase of Treg.

### DCR-5 Can Induce DCreg Phenotype on Purified FL-DC Through Binding to CD83

**Figure 8:**
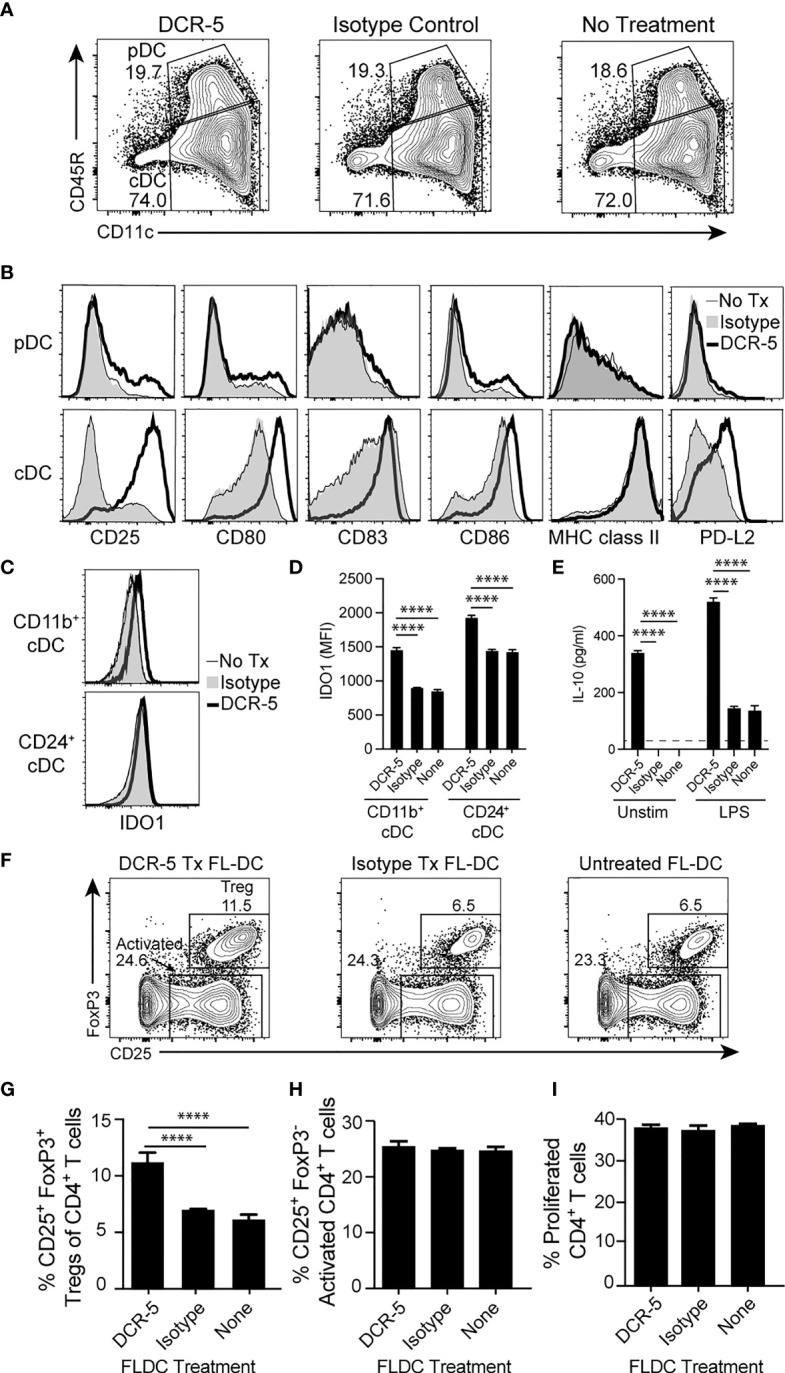
DCR-5 binding to FL-DC population induces DCReg. **(A)** Flow cytometry plots showing gating of cultured B6 FL-DC cDC and pDC subsets after overnight culture with 20 µg/ml DCR-5, isotype control or no treatment and **(B)** histograms comparing their expression of maturation markers. **(C)** Representative histograms and **(D)** graph displaying flow cytometric intracellular expression (MFI) of IDO1 in FL-DC cDC subsets treated as above (n = 3). **(E)** Concentration of IL-10 in supernatants (n = 3) of FL-DC cultured for 48 h with 20 µg/ml DCR-5, isotype control or no antibody in the absence or presence of LPS stimulation. Dotted line shows limit of detection. **(F)** Representative contour plots and **(G)** graph (n = 3) of H-2K^d+^CD4^+^CD25^+^FoxP3^+^ Treg generated from purified naïve BALB/c splenic T cells cultured at an 8:1 ratio for 3 days with washed B6 FL-DC after overnight treatment with DCR-5, isotype control or no treatment. **(H)** Graphs comparing CD25^+^ FoxP3^−^ T cells and **(I)** proliferated CFSE labeled CD4^+^ T cells in the same experiment. All groups compared by one-way ANOVA. ****p < 0.0001.

To determine whether binding of DCR-5 to CD83 on DC was sufficient to induce a DCreg phenotype in the absence of other immune cells that mediate cytotoxicity, we generated a population of FL-DC from B6 bone marrow (consisting of >90% cDC and pDC). These were treated with DCR-5 or isotype control antibodies overnight and assessed for upregulation of DC maturation markers. While DCR-5 treatment caused notable changes to the surface phenotype of cDC but not pDC from these cultures, their proportions were not affected (**Figures 8A** **, B**). DCR-5 treatment of FL-DC in the absence of other cells led to most cDC expressing CD83 compared to isotype and untreated counterparts where some CD83^-/lo^ cDC were present; although a proportion of control treated FL-DC expressed higher levels of CD83 than DCR-5 treated cDC (**Figure 8B**). Akin to observations in MLR and the *in vivo* experiments, DCR-5 treatment caused upregulation of CD80 and CD86 on cDC, which coincided with increases in CD25 and PD-L2 DCreg markers. Analogous DCR-5 mediated induction of these markers were seen in CD11b^+^ and CD24^+^ subsets corresponding to CD8^-^ and CD8^+^ cDC (data not shown). Intracellular IDO1 expression was significantly increased by DCR-5 in both subsets (**Figures 8C** **, D**). Secretion of IL-10 was upregulated by exposure of FL-DC to DCR-5 in the absence or presence of maturation signals by LPS (**Figure 8E**). When DCR-5 treated FL-DC were washed and re-cultured with allogeneic BALB/c T cells, we saw a significantly increased capacity to induce Treg compared to control treated FL-DC, consistent with DCreg function (**Figures 8F** **, G**). This was not due to greater T cell activation as no change was observed in the percentage of activated (CD25^+^ FoxP3^-^) or proliferated CD4^+^ T cells in the cultures (**Figures 8H** **, I**). These findings suggest that DCR-5 binding to CD83 on DC can directly induce maturation of DCreg which efficiently produce Tregs.

### Optimized Dose of DCR-5 Reduces Severity of CIA

**Figure 9:**
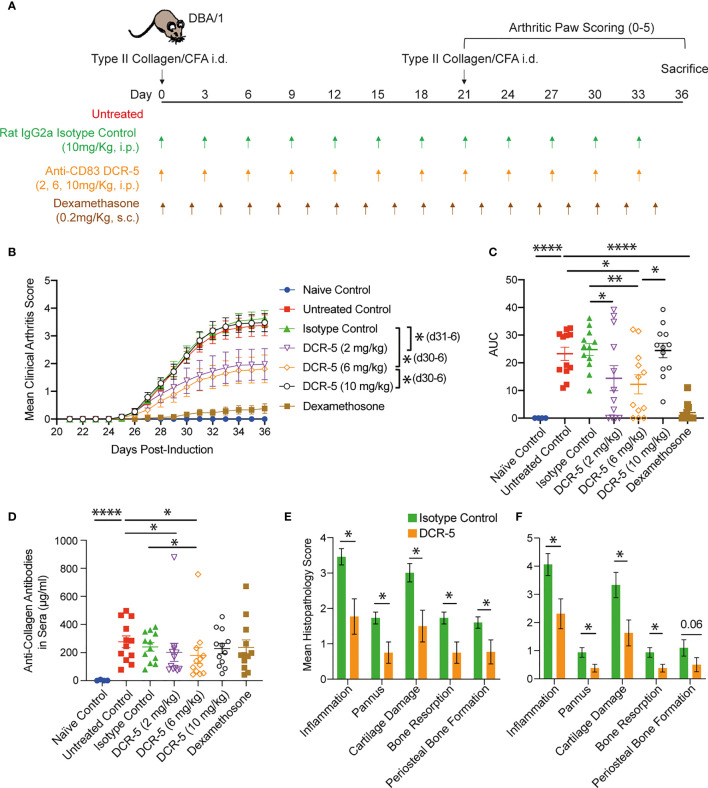
Assessment of DCR-5 efficacy in CIA model. **(A)** Schema of CIA model and treatment regimes in five cohorts of 12 DBA/1 female mice. An additional four mice were not immunized with type II collagen in CFA as a naïve control cohort. **(B)** Mean arthritis clinical score per paw over time and **(C)** area under the curve (AUC) for each individual mouse (lines mark mean). Statistically significant differences at individual timepoints (in parentheses) and AUC were performed using one-way ANOVA. Significantly different timepoints outlined in parentheses. **(D)** Anti-collagen antibodies in serum at end of experiment were determined by ELISA. Statistical comparison of samples was determined by one-way ANOVA. **(E)** Mean paw and **(F)** knee score for each histopathology parameter in 6 mg/kg DCR-5 group versus the isotype control treated group at sacrifice. Statistical comparison of samples was determined by Mann–Whitney test. *p < 0.05, **p < 0.01 and ****p < 0.0001.

To determine whether anti-CD83 induced changes in the studies above would provide beneficial responses relevant for the treatment of autoimmune diseases, we assessed whether DCR-5 could reduce the severity of CIA developed by DBA/1 mice using the treatment regimens outlined in **Figure 9A**. Groups treated with different doses of DCR-5 (2, 6, and 10mg/kg) every three days throughout the study were compared to untreated, isotype control treated (10 mg/kg) and dexamethasone (0.2 mg/kg) treated groups. Weights were similar between DCR-5, isotype control or untreated groups through the study (**Supplementary Figure 4A**). However, mice treated with 2 and 6 mg/kg DCR-5 developed significantly lower clinical arthritic paw scores compared to mice treated with the isotype control or untreated animals (**Figures 9B** **, C**). The protective effect was lost in the 10 mg/kg DCR-5 treated group. Consistently, 6 mg/kg DCR-5 treatment resulted in a significant decrease in serum anti-collagen antibodies compared to the isotype control and untreated groups, with a more modest decrease in 2 mg/kg and no effect from 10 mg/kg treatment (**Figure 9D**). Histopathological analysis of knees and ankles was performed on the isotype control and 6 mg/kg doses and showed significant amelioration of inflammation, pannus, cartilage damage, bone resorption, and periosteal bone formation from 6 mg/kg DCR-5 treatment compared to the isotype control (**Figures 9E, F** and **Supplementary Figure 4B**). Treatment with DCR-5 up to 6 mg/kg was therefore effective in alleviating disease in the CIA model.

### DCR-5 Treatment Depleted CD83^+^ DC and Induced Treg in CIA Model

**Figure 10:**
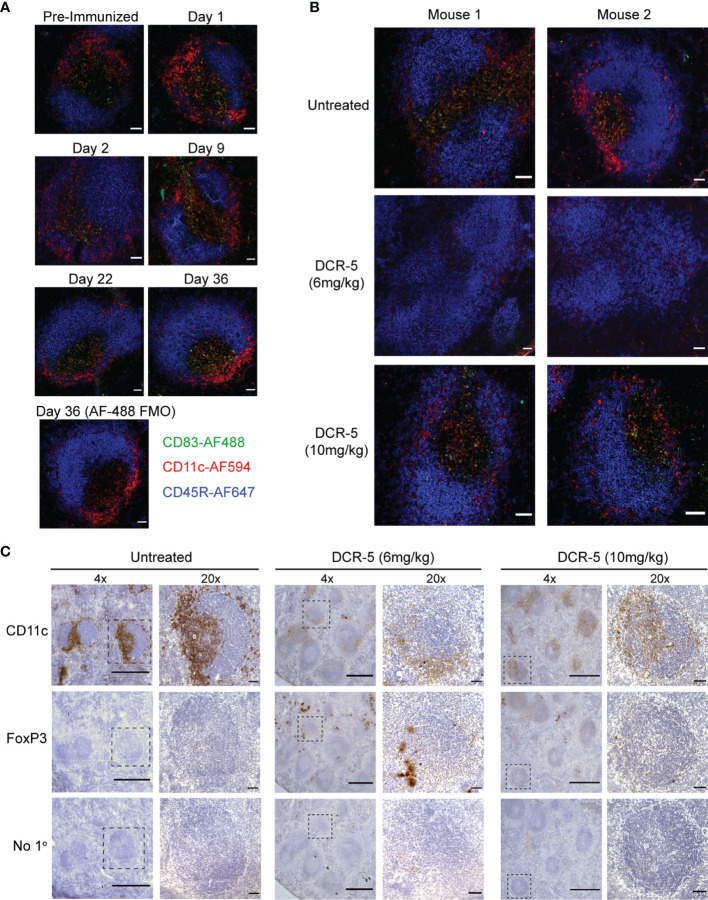
DCR-5 treatment decreases CD11c staining and increases FoxP3 staining in spleens of CIA mice. **(A, B)** Immunofluorescence histology of splenic follicles showing CD83 detected with biotinylated Michel-19 clone together with CD11c and B220 marking DC and B cells, respectively. **(A)** Shows representative ×20 images from one of two DBA/1 mice euthanized pre-immunization or on the indicated days post-immunization of collagen. A control stain omitting the anti-CD83 primary antibody but including the Streptavidin-AF488 secondary is shown. Scale bars mark 50 µm. **(B)** Shows representative ×20 images of splenic follicles from two of four mice from untreated, 6 mg/kg or 10 mg/kg DCR-5 treated groups described in **Figure 9**. Scale bars mark 50 µm **(C)** Immunohistochemistry of adjacent spleen sections stained with biotinylated CD11c, FoxP3 antibodies or secondary streptavidin-HRP only. Representative ×4 image and marked inset at ×20 from one of four mice in untreated, 6 and 10 mg/kg DCR-5 treated groups are shown. Scale bars mark 500 and 50 µm in ×4 and ×20 photographs, respectively.

Increased immunohistological CD83 staining in areas of lymphoid follicles that co-stain with CD11c (marking DC), but not CD45R (marking B cells), was observed in spleen sections of untreated DBA/1 mice euthanized at different timepoints during CIA development (**Figure 10A**). Analysis of spleen sections from 6 mg/kg DCR-5 treated compared to isotype control animals at the end of the CIA model (d36) revealed a clear decrease in CD11c and CD83 staining within follicles, indicative of specific depletion of DC (**Figure 10B**). Depletion of CD83^+^ DC was less evident in spleens of 10 mg/kg DCR-5 treated mice. A similar decrease in CD11c staining in splenic follicles of 6 mg/kg DCR-5 treated mice was observed using immunohistochemistry methods (**Figure 10C**). DC depletion was not as marked in spleens of 10 mg/kg DCR-5 treated mice. In contrast, FoxP3 staining was increased in adjacent sections of 6 mg/kg DCR-5 treated mice compared to isotype control mice, indicating Treg induction (**Figure 10C**). Interestingly, despite poor disease control, limited FoxP3 staining was also induced in spleens of the 10 mg/kg DCR-5 group. Protection from arthritis in mice treated with 6 mg/kg DCR-5 is, therefore, associated with depletion of DC and induction of Treg in spleens, with these effects being less marked in 10 mg/kg DCR-5 treated mice.

### Anti-Drug Antibodies (ADA) Elicited by DCR-5

To examine whether disparity in efficacy of DCR-5 at 6 and 10 mg/kg doses was caused by differences in anti-drug antibodies elicited by the treatments, we examined the sera collected at the end of the study (d36) from CIA groups for anti-DCR-5 antibodies *via* an anti-DCR-5 ELISA (**Supplementary Figure 4C**). High levels of anti-DCR-5 antibodies were detected in the sera from 2, 6, and 10 mg/kg DCR-5 treated animals by d36 with no significant differences between the groups. Spiking the DCR-5 treated mouse serum samples with DCR-5, reduced binding in the ELISA indicative of anti-DCR-5 specificity. Interestingly, despite sharing immunoglobulin constant regions with DCR-5, treatment with 10 mg/kg isotype control did not stimulate measurable production of antibodies capable of binding to DCR-5.

## Discussion

Targeting mature DC for depletion with anti-CD83 antibodies is a unique strategy for achieving specific immune modulation. Depletion of maturing DC prevents the ability to prime an effector T cell response while retaining the immature (CD83^-^) DC that promotes T cell self-tolerance. As T cells are not the prime target, this treatment retains memory T cells specific for viral or tumor antigens, which are less reliant on DC for activation while accessory APCs such as B cells, macrophages, and non-haematopoietic cells can still activate an effector response (31, 32). Preserving memory T cells would provide a clinical advantage to patients by maintaining some immunity, preserving childhood vaccination responses, and limiting immune suppression.

While our human anti-human CD83 mAb 3C12C has shown the ability to deplete mature cDC and limit T cell responses in *in vitro* MLR experiments and xenogeneic graft-versus-host disease models (21), the ability to test the efficacy of this reagent in other inflammatory conditions such as autoimmune disease was limited by the dearth of animal models available to test human antibodies. The DCR-3 and DCR-5 rat mAbs were produced to mimic anti-CD83 mediated immune suppression in mice, to better understand its mechanism of action and test further indications for this therapeutic strategy in the plethora of inflammatory disease models developed in this species.

DCR-3 and DCR-5 exhibited greatest binding to the surface of mature cDC following activation, with less binding seen on activated B cells and other APC. However, DCR-3 and DCR-5 showed low levels of binding to CD83 on activated Treg compared to the Michel-19 antibody. While this may be attributed to differences in affinity, the similar degree of binding by the antibodies to CD83 protein by ELISA and on the DC surface by flow cytometry would suggest that different CD83 isoforms on distinct cell populations contribute to the binding disparity. This was corroborated in our immunoprecipitation studies with DCR-5 and Michel-19, where common, but also distinct isoforms of CD83 were detected on the surface of mouse DC compared to B cells. Moreover, in our human studies (16), the HB15a mouse anti-human CD83 mAb clone bound preferentially to cell surface CD83 and less to intracellular CD83, with the reverse noted for the HB15e clone. MLR assays demonstrated that the DCR-5 IgG2a mAb had the ability to deplete CD83^+^ cDC and inhibit T cell proliferation, while the DCR-3 IgG2b mAb did not. While rat IgG2a antibodies are more effective at activating rat NK cell ADCC than rat IgG2b (33, 34), in mice, rat IgG2b showed significantly greater capacity to induce ADCC than rat IgG2a (35). The lack of functional activity by DCR-3 is, therefore, surprising, and again highlights the importance of the CD83 epitope targeted for functional activity.

cDC that were not depleted by DCR-5 in the MLR assay expressed low to absent levels of CD83 but upregulated costimulatory molecules CD80 and CD86 together with the regulatory molecules PD-L2, CD25 and the IDO1 enzyme, indicative of DCreg maturation (4, 30, 36, 37). In addition, significant increases in IL-10 production and inhibition of IL-2 production were observed in DCR-5 treated MLR cultures. IL-10 production was induced in DC and Tcon in MLR. This is most likely due to direct and indirect effects of DCR-5, respectively, given that DC and not T cells were found to express CD83 in these cultures. DCR-5 was also shown to induce IL-10 production by FL-DC cultures. IL-10 is an important immunoregulatory cytokine that induces, and is produced by, DCreg and Treg (4, 38). CD25 expressed on the surface (and released in soluble form) by DCreg and also Treg acts to sequester available IL-2 to suppress conventional T cell proliferation (30, 38, 39). Expression of PD-L1 and PD-L2 by DCreg can induce deletion, anergy or Treg differentiation of interacting T cells expressing PD-1 (4). DCreg production of kynurenine, produced by tryptophan metabolism *via* the IDO1 enzyme, is a potent inducer of Treg differentiation upon binding to the aryl hydrocarbon receptor on T cells (4, 37, 40).

*In vivo* studies provided further insight into the function of DCR-5. I.p. injection of DCR-5 into mice showed that the DCR-5 antibody had the capacity to deplete splenic CD83^hi^MHCII^hi^ cDC populations *in vivo*. Cross-presenting CD8^+^ cDC were particularly susceptible to DCR-5 depletion *in vivo* compared to CD8^−^ cDC. While higher expression of CD83 was seen in LPS and CpG stimulated CD8^+^ DC compared to CD8^-^ cDC, the majority of CD8^+^ cDC did not express CD83 in untreated or isotype treated mice. It is possible that exposure to DCR-5 may cause upregulation of CD83 in CD8^+^ cDC. Akin to the MLR, DCR-5 induced the population of CD83^lo^ CD80^hi^/86^hi^ cDC *in vivo* that upregulated DCreg markers including CD25, PD-L2 and PD-L1. The correlation found between the CD80^hi^/86^hi^ cDC population and Treg in DCR-5 injected mice was indicative of DCreg. We found that induction of a CD80^hi^/86^hi^ PD-L2^+^CD25^+^IDO1^+^ phenotype with enhanced IL-10 producing capacity reminiscent of DCreg could be achieved by culturing DCR-5 with FL-DC alone. Moreover, this population had a heightened capacity to promote Treg differentiation, confirming DCreg function. Therefore, in addition to depletion of cDC, DCR-5 can drive DC maturation to a regulatory phenotype *via* binding to CD83. A previous study by Bates et al. (41) showed that ligation of membrane CD83 on human monocyte derived DC by anti-CD83 antibodies could mimic the immunoregulatory signals mediated by soluble CD83. Soluble CD83 can bind homotypically to membrane CD83 on DC resulting in inhibition of the p38 MAPK pathway (41) and induction of DCreg (42–46). While anti-CD83 antibodies have the potential to neutralise soluble CD83 regulatory activity (22), our data indicates that this may be compensated by antibody binding to membrane CD83. DCR-5 mediated apoptosis of mature DC by ADCC also has the potential to induce a DCreg phenotype when detected by bystander DC (47). This would fit with the negative correlation observed between the extent of DCR-5 mediated DC depletion and induction of the CD80^hi^/86^hi^ DC population. The extent to which these or other mechanisms contribute to DCreg generation by anti-CD83 mAb will be important to determine.

The relevance of our findings to human disease remains to be tested. In mice, immune populations expressing low levels of surface CD83 upon activation, namely, B cells, pDC, and Treg seem to be spared from DCR-5 mediated depletion. In contrast, the 3C12C human therapeutic mAb, which targets human and nonhuman primate CD83 has the capacity to deplete activated B cells both in human PBMC xenogeneic mouse models and non-human primates (22, 48). Unlike 3C12C that underwent affinity maturation using a light chain shuffling procedure (21) to select an antibody with higher affinity for human CD83, DCR-5 has not undergone this procedure and displays a 500-fold lower affinity for mouse CD83. This can contribute to their targeting differences. Despite reported differences between CD83 on T cells between mice and humans, neither 3C12C or DCR-5 antibody substantially depletes T cells, including Treg. While human Treg express minimal surface CD83 (16, 49), surface CD83 is found on a proportion of activated conventional T cells in humans and mice (29, 49–52). DCR-5 did not reduce conventional CD4^+^ or CD8^+^ T cells when injected into naïve mice. The lack of depletion is consistent with either the paucity of activated T cells in naïve mice or expression of a different isoform of CD83 on activated T cells. CD83 was not detected on T cells undergoing allogeneic proliferation in our MLR assay nor did we see marked CD83 expression on non-Treg T cells after CD3/CD28 activation. It is important to note that conventional T cells were not reduced post DCR-5 treatment in mice, indicating that the antibody is not likely to target CD83^+^ thymic epithelial cells, which are essential for positive selection of T cells (18, 53).

DC in RA patients are central to the activation of autoreactive CD4^+^ and CD8^+^ T cells that drive effector mechanisms including autoantibody production, and for the recruitment of other inflammatory cells that induce joint pathology (9, 10). In mice, administration of mature DC pulsed with collagen can initiate arthritis, suggesting that these cells are sufficient to drive disease pathogenesis (54). Alternately, administration of autoantigen presenting DC that are prevented from undergoing maturation, or differentiated into a regulatory phenotype, provide significant protection from arthritis (11–13). The main mechanism underlying this protection was the ability of transfused tolerogenic DC to enhance generation of Treg, which are potent at countering arthritogenic autoimmunity. Our study showed that DCR-5 treatment could deplete mature CD83^+^ DC and augment the number of Treg in the CIA model, mechanisms that are likely to be essential to explaining our observations of reduced anti-collagen antibody titers, improved clinical scores, and preserved joint structure. Interestingly, the effect appeared to be dose dependent with up to 6 mg/kg dose providing optimal disease protection, while the higher 10 mg/kg dose was ineffective. Examination of spleens from treated animals indicated that the 10 mg/kg DCR-5 dose did exhibit some capacity to deplete DC and induce Treg in mice, but this was not as marked as in spleens from mice receiving the 6 mg/kg dose. One reason for the difference may be the onset and presence of ADA, as rat IgG2a antibodies can be highly immunogenic in mice, particularly when targeted to DC (55). All DCR-5 regimens induced high titers of ADA in serum by the end of the model. In most cases, higher doses of cross-species mAb in pre-clinical murine models result in higher risk of ADA (56, 57). Elevated levels of drug-ADA immunocomplexes also have the capacity to drive T cell independent B cell activation, further enhancing the ADA response (58, 59). It is conceivable that in our study the higher 10 mg/kg DCR-5 dose generates higher titers of ADA and immunocomplexes at earlier timepoints during the disease model, thereby shortening the therapeutic window. The 6 mg/kg dose could provide a more optimal balance of drug efficacy and ADA generation. The potential for ADA is reduced when treating humans with human mAb, however, assessment of ADA will be necessary when evaluating the 3C12C mAb in clinical studies. Another possibility for the lack of efficacy in the 10 mg/kg dose is that that higher doses of DCR-5 could target cells with more limited CD83 expression, namely, DCreg and Treg, leading to depletion of these cells and resulting in no observable pharmacodynamic effect. Our preliminary DCR-5 dose titration study in B6 mice did not reveal dose dependent differences in the type of immune populations that were depleted (e.g., B cells or pDC) or differences in Treg induction using higher doses (e.g., 250–500 µg). It should be noted though that this dose titration study administered a single dose under non-inflammatory conditions. Careful consideration of dose will be required to achieve optimal anti-CD83 immune modulation in patients.

**Figure 11:**
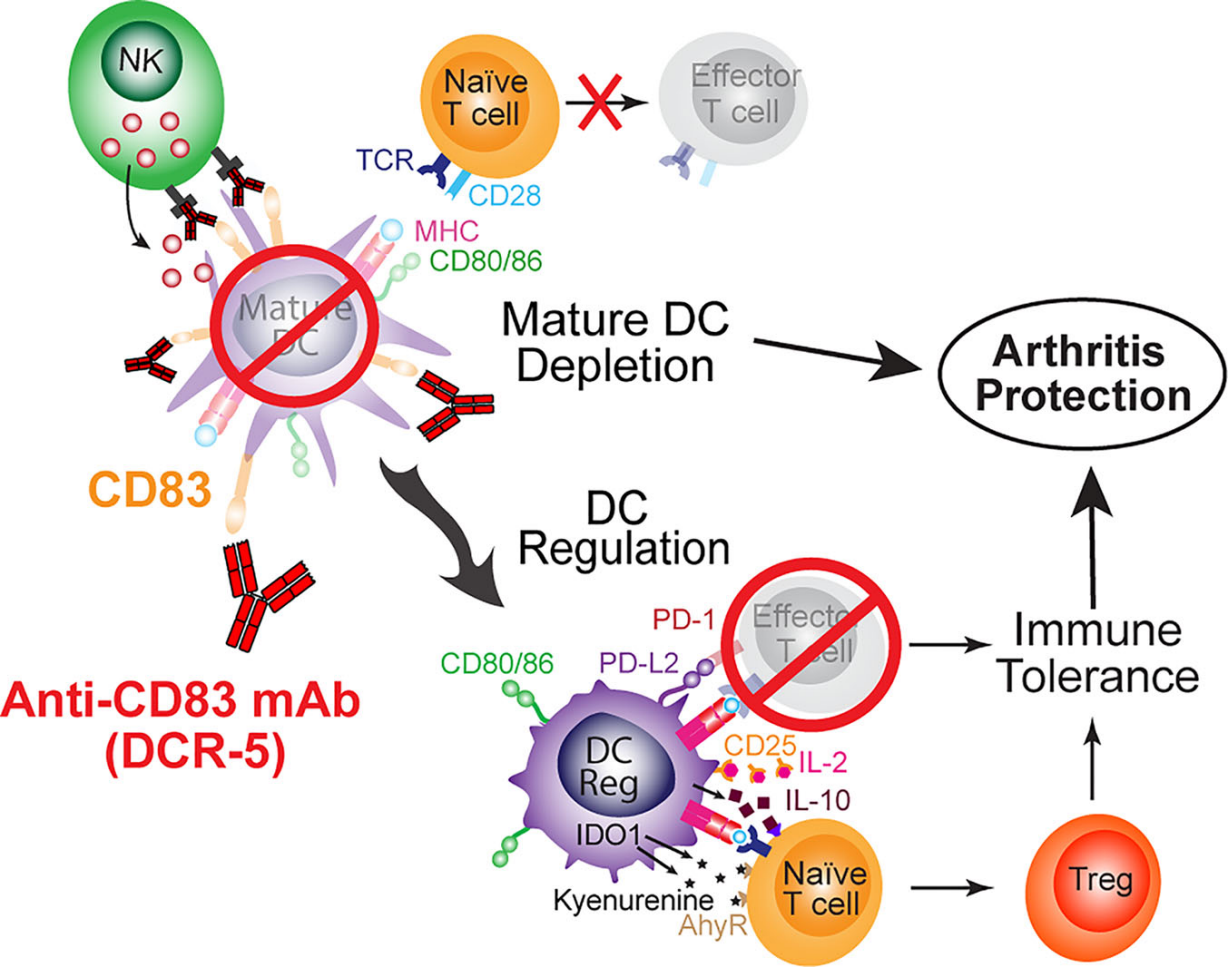
Schematic of mechanisms of anti-CD83 mAb mediated immune suppression leading to protection from arthritis.

In conclusion, we show here for the first time that depletion of mature DC by ligation of CD83 offers a unique mechanism for immunomodulation (**Figure 11**). The mechanism appears to involve the ability of the anti-CD83 antibody to deplete CD83^hi^ DC, reducing antigen presentation by activated mature DC, upregulation of DCreg and the subsequent enhanced development of Treg. By using the CIA model, we demonstrate that these properties translate to effectiveness in a model that fits the relevant biology and is a common animal model for rheumatoid arthritis. The biology of anti-CD83 antibodies has potential in the treatment of autoimmune, inflammatory or transplant related diseases. Further animal models, including murine models using DCR-5 and nonhuman primate models using the human/primate antibody (3C12C) will address the pre-clinical and mechanistic studies to understand how this treatment could benefit patients with these diseases.

## Data Availability Statement

The original contributions presented in the study are included in the article/**Supplementary Material**. Further inquiries can be directed to the corresponding author.

## Ethics Statement

The animal studies were reviewed and approved by the Bolder Biopath Ethics Committee and the Sydney Local Health District Animal Welfare Committee.

## Author Contributions

PS designed research studies, conducted experiments, acquired, and analysed data and wrote the manuscript. FK, AR, W-HH, T-HL, and H-TC conducted experiments, acquired, and analyzed data. XJ, HR, and DB contributed to the design of research studies, analysis of data and reviewing the manuscript. GC contributed to the design of research studies, analysis of data and writing of the manuscript. All authors listed have made a substantial, direct, and intellectual contribution to the work and approved it for publication.

## Funding

This work was funded by a Translational Program Grant (2017/TPG002) from the Cancer Institute New South Wales. Also, Sponsored Research was received from Kira Biotech Pty Ltd. W-HH received a University of Sydney, Enid Ng Fellowship and H-TC received a University of Sydney, Research Training Program (RTP) Stipend Scholarship.

## Conflict of Interest

PS, XJ, T-HL, AR and FK work for the Dendritic Cell Research laboratory which received sponsored research funding from Kira Biotech to undertake work quoted in this paper. HR derived a salary from Kira Biotech and has equity in the company. DB is a Director of Kira Biotech Pty Ltd. and has equity in the company. GC is a Non-Executive Director of Kira Biotech Pty Ltd. and Dendrocyte Pty Ltd. which has equity in Kira Biotech Pty Ltd. GC is Group Leader of the Dendritic Cell Research laboratory which received sponsored research funding to undertake work quoted in this paper. W-HH and H-TC declare no conflicts of interest.

## Publisher’s Note

All claims expressed in this article are solely those of the authors and do not necessarily represent those of their affiliated organizations, or those of the publisher, the editors and the reviewers. Any product that may be evaluated in this article, or claim that may be made by its manufacturer, is not guaranteed or endorsed by the publisher.

## Abbreviations

ADA, anti-drug antibodies; ADCC, antibody dependent cell cytotoxicity; AF, Alexa Fluor; APC, antigen presenting cell; cDC, conventional dendritic cell; CFSE, carboxyfluorescein succinimidyl ester; CIA, collagen induced arthritis; CTV, cell trace violet; DC, Dendritic Cells; DCreg, regulatory dendritic cell; FL-DC, Flt-3L cultured bone marrow DC; i.p., intra-peritoneal; mAb, monoclonal antibody; MHC, major histocompatibility complex; MLR, mixed leukocyte reaction; pDC, plasmacytoid dendritic cell; RA, rheumatoid arthritis; Tcon, conventional T cell; Treg, regulatory T cell.
