# Supplementary material for: Anti-Mouse CD83 Monoclonal Antibody Targeting Mature Dendritic Cells Provides Protection Against Collagen Induced Arthritis
Source: Front Immunol. 2022 Feb 10;13:784528. doi: 10.3389/fimmu.2022.784528 (PMC8866188; doi:10.3389/fimmu.2022.784528)
Supplement: Supplementary file 1 [file DataSheet_1.pdf]

SUPPLEMENTARY MATERIAL

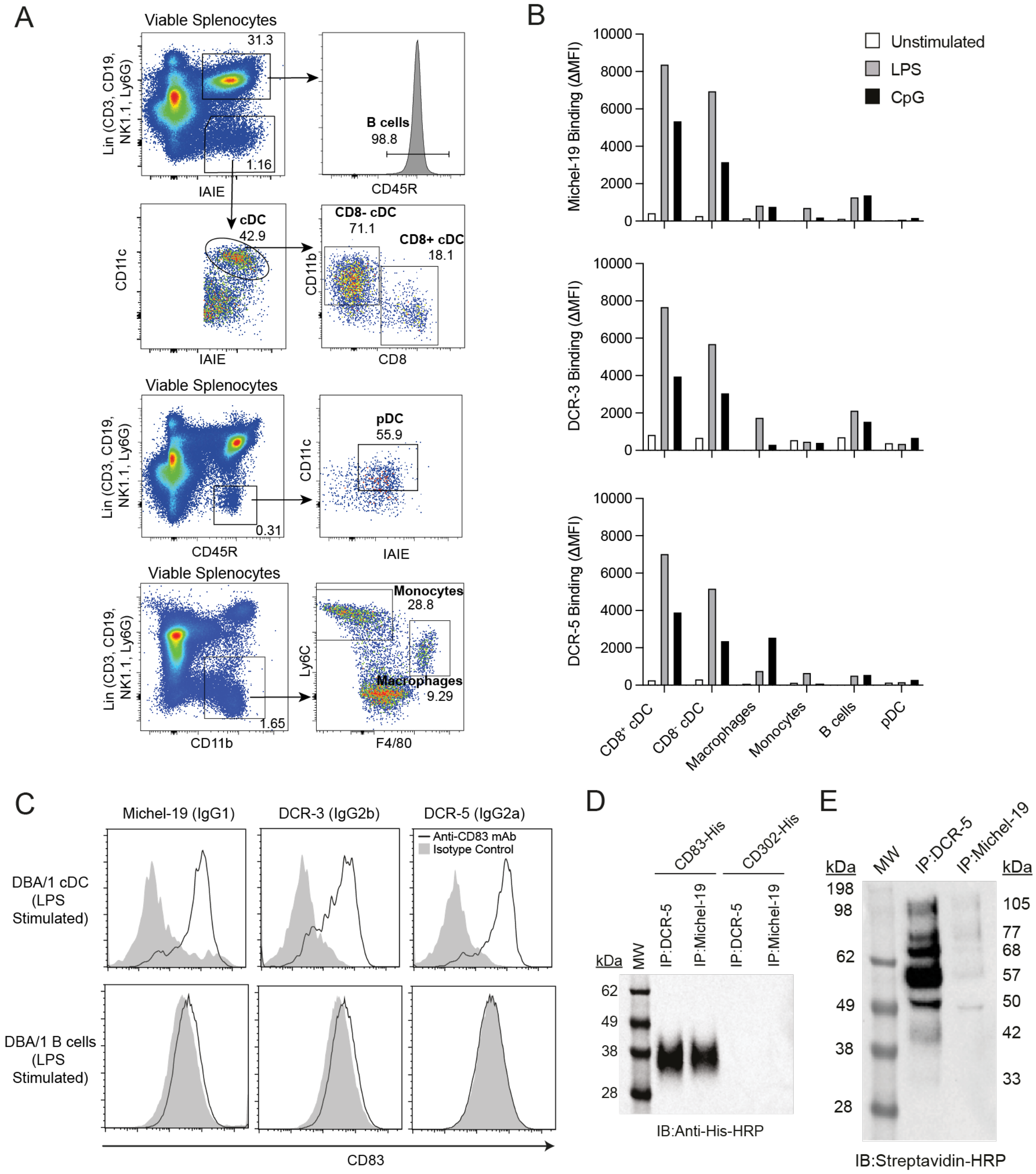

Supplementary Figure 1 | Rat anti-mouse CD83 antibody binding on APC from the DBA/1 mouse strain and T cell subsets. **(A)** Gating strategy for APC subsets in spleen. **(B)** ΔMFI of binding with 20µg/ml Michel-19, DCR-3 or DCR-5 antibody minus that of respective isotype in indicated APC subsets cultured overnight at 37°C with 1µg/ml LPS, 1.25µM CpG-ODN 2395 or left unstimulated at 4°C. **(C)** Binding with 20µg/ml Michel-19, DCR-3 or DCR-5 antibody to the surface of gated DC (Lin^- $IAIE^+$ $CD11c^+$) and B cells (Lin^+ $IAIE^+$ $CD45R^+$) from DBA/1 mouse splenocytes cultured overnight with 1µg/ml LPS. **(D)** Immunoprecipitation (IP) of recombinant mouse CD83-HisTag or negative control mouse CD302-HisTag with DCR-5 or Michel-19 coated beads. Captured proteins were separated by reduced SDS-PAGE and immunoblotted (IB) with anti-His Tag-HRP. Molecular weight (MW) standards shown in first lane. **(E)** Immunoprecipitate (IP) of lysates from surface biotinylated LPS-stimulated FL-DC with DCR-5 or Michel-19 mAb coated beads. Captured proteins were separated by reduced SDS-PAGE and immunoblotted (IB) with streptavidin-HRP to detect biotinylated CD83 variant bands. Molecular weight standards shown in lane 1 and estimated band sizes in lanes 2-3 shown on the right.

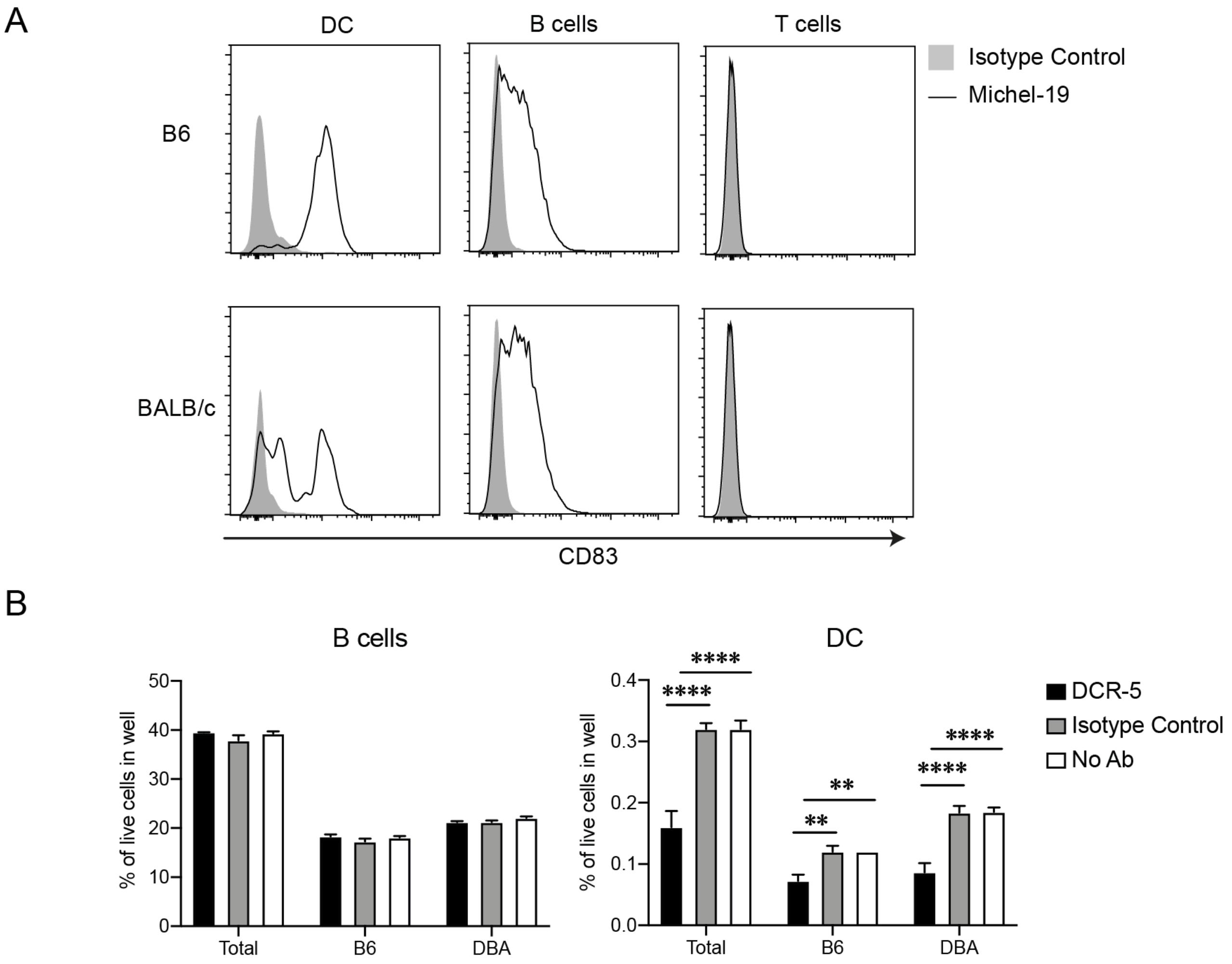

Supplementary Figure 2 | Anti-CD83 in MLR assays. **(A)** CD83 expression was detected on untreated B6 or BALB/c DC (Lin$^-$IAIE$^+$CD11c$^+$), B cell (Lin$^+$IAIE$^+$B220$^+$) and T cells (Lin$^+$IAIE$^-$CD4$^+$ or CD8$^+$) after overnight culture of MLR using the Michel-19 mAb. **(B)** Total, B6 (CD45.1 congenic) and DBA/1 B cell and DC depletion in B6 x DBA/1 MLR after overnight culture with DCR-5, isotype control or no antibody (n=3). Statistical comparison by one-way ANOVA. ** $p < 0.01$ and **** $p < 0.0001$.

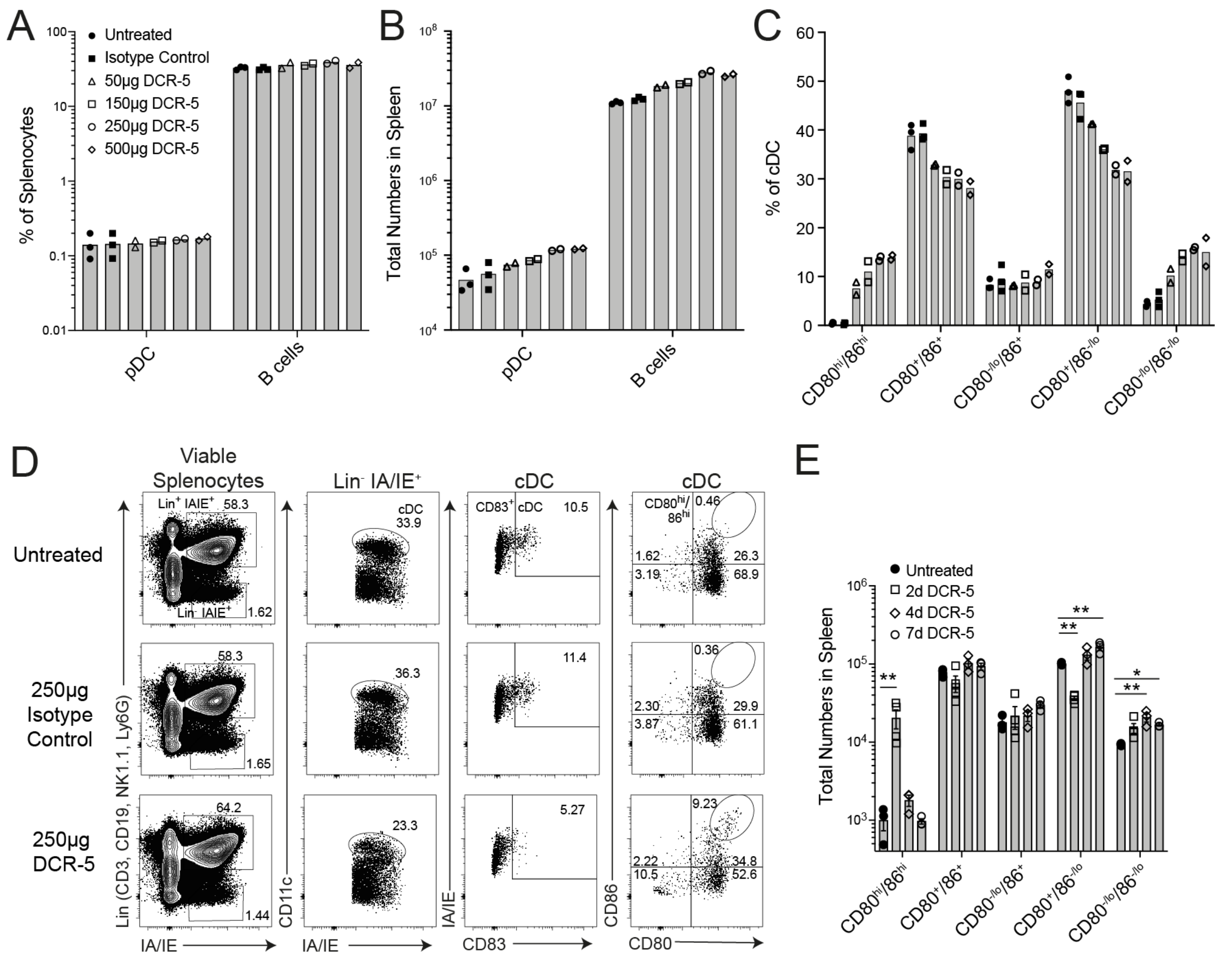

Supplementary Figure 3 | *In vivo* anti-CD83 treatment. Spleens from B6 mice were analysed by flow cytometry 48h after i.p. injection of indicated doses of DCR-5 or 250µg of isotype control or no treatment. **(A)** Frequency and **(B)** total numbers of B cells and pDC (as gated in Supplementary Figure 1A) in spleens of treated or untreated mice shown. **(C)** Frequency of CD80$^{-/lo}$/CD86$^{-/lo}$, CD80$^{-/lo}$/CD86^{+}, CD80^{+}/CD86$^{-/lo}$, CD80^{+}/CD86^{+} cDC shown in quadrants and CD80^{hi}/CD86^{hi} cDC in oval (as gated in Figure 4A – last column) in all treated or untreated mice. All groups except CD80^{-}CD86^{+} exhibited p<0.0001 statistical difference when comparing combined DCR-5 treated mice to isotype or untreated animals (two-way ANOVA). **(D)** Comparison of cDC depletion in spleens of DBA/1 mice treated i.p. with 250µg DCR-5 or isotype control for 48h or untreated. Representative plots from one of three mice shown. **(E)** Total number of CD80/86 cDC populations (as gated in Figure 4A – last column) in spleens of B6 mice after no treatment or 2, 4 or 7d post-treatment with 150µg DCR-5 g i.p. Statistical differences to untreated group determined by two-way ANOVA. * $p < 0.05$ and ** $p < 0.01$.

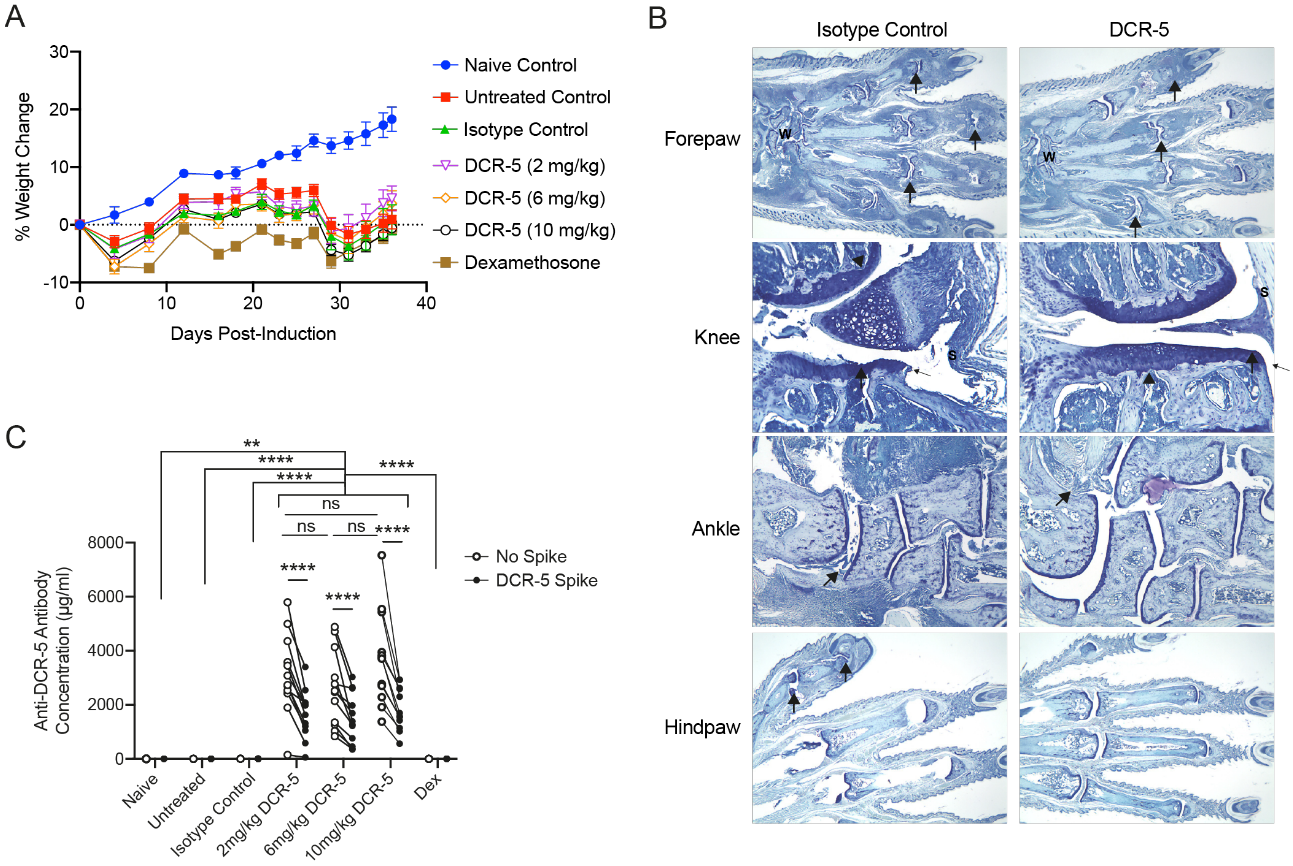

Supplementary Figure 4 | Anti-CD83 treatment in CIA model. **(A)** Weights of mice in the indicated treatment groups during the CIA experiment described in Figure 9A. No significant differences in weights at individual timepoints or AUC between untreated, isotype or DCR-5 treated groups (one-way ANOVA). **(B)** Representative histopathology of forepaw, knees, ankle and hind paw of 6mg/kg DCR-5 or 10mg/kg isotype control treated mice with approximate mean score on d36 of the CIA model. Thick arrows indicate affected joints, thin arrows show pannus, arrowheads show bone resorption, W identifies wrist and S marks inflammation. **(C)** Concentrations of anti-drug antibodies in end of experiment sera of mice from all CIA groups in Figure 4 were determined by ELISA. Specificity was confirmed in serum samples spiked with 1µg/ml DCR-5 and incubated for 20 minutes at 37°C before being analysed. Comparison between non-spiked and spiked serum samples was performed by paired t-test. Comparison of non-spiked samples between groups was performed by one-way ANOVA. ** $p < 0.01$ and **** $p < 0.0001$.
